# Complexes of Vesicular Stomatitis Virus Matrix Protein with Host Rae1 and Nup98 Involved in Inhibition of Host Transcription

**DOI:** 10.1371/journal.ppat.1002929

**Published:** 2012-09-27

**Authors:** Karishma R. Rajani, Elizabeth L. Pettit Kneller, Margie O. McKenzie, David A. Horita, Jeff W. Chou, Douglas S. Lyles

**Affiliations:** 1 Department of Biochemistry, Wake Forest School of Medicine, Winston Salem, North Carolina, United States of America; 2 Department of Biostatistical Sciences, Division of Public Health Sciences, Wake Forest School of Medicine, Winston Salem, North Carolina, United States of America; Harvard Medical School, United States of America

## Abstract

Vesicular stomatitis virus (VSV) suppresses antiviral responses in infected cells by inhibiting host gene expression at multiple levels, including transcription, nuclear cytoplasmic transport, and translation. The inhibition of host gene expression is due to the activity of the viral matrix (M) protein. Previous studies have shown that M protein interacts with host proteins Rae1 and Nup98 that have been implicated in regulating nuclear-cytoplasmic transport. However, Rae1 function is not essential for host mRNA transport, raising the question of how interaction of a viral protein with a host protein that is not essential for gene expression causes a global inhibition at multiple levels. We tested the hypothesis that there may be multiple M protein-Rae1 complexes involved in inhibiting host gene expression at multiple levels. Using size exclusion chromatography and sedimentation velocity analysis, it was determined that Rae1 exists in high, intermediate, and low molecular weight complexes. The intermediate molecular weight complexes containing Nup98 interacted most efficiently with M protein. The low molecular weight form also interacted with M protein in cells that overexpress Rae1 or cells in which Nup98 expression was silenced. Silencing Rae1 expression had little if any effect on nuclear accumulation of host mRNA in VSV-infected cells, nor did it affect VSV's ability to inhibit host translation. Instead, silencing Rae1 expression reduced the ability of VSV to inhibit host transcription. M protein interacted efficiently with Rae1-Nup98 complexes associated with the chromatin fraction of host nuclei, consistent with an effect on host transcription. These results support the idea that M protein-Rae1 complexes serve as platforms to promote the interaction of M protein with other factors involved in host transcription. They also support the idea that Rae1-Nup98 complexes play a previously under-appreciated role in regulation of transcription.

## Author Summary

All viruses have mechanisms to suppress or evade host antiviral responses. These mechanisms are critical for viral pathogenicity. Vesicular stomatitis virus (VSV) suppresses antiviral responses by global inhibition of host gene expression mediated by the viral matrix (M) protein. M protein interacts with the host protein Rae1 in a complex with the nucleoporin Nup98. It had been thought that interaction of M protein with Rae1 blocks nuclear-cytoplasmic mRNA transport. However, other data show that Rae1 is not essential for mRNA transport. With this discrepancy in mind, we re-examined the interaction of M protein with Rae1 and Nup98 and the level of host gene expression in which they are involved. A key result was that silencing Rae1 expression did not affect host gene expression, but instead increased cellular resistance to inhibition by M protein. Furthermore, silencing Rae1 expression primarily affected the inhibition of host transcription with no significant effect on nuclear accumulation of mRNA. These results support a model in which Rae1 serves as a “platform” to promote interaction of M protein with cellular targets involved in host transcription. This illustrates a general principle that viral proteins can have multiple cellular effects by interacting with host proteins that are themselves multi-functional.

## Introduction

The antiviral responses mounted by virus-infected cells include potent mechanisms to prevent virus replication. Thus, in order for viruses to effectively propagate, most viruses have developed mechanisms to inhibit or evade these host antiviral responses. Many RNA viruses that replicate in the cytoplasm suppress antiviral responses by inhibiting host nuclear functions, such as transcription and nuclear-cytoplasmic transport. Vesicular stomatitis virus (VSV) is a widely studied prototype of the negative strand RNA viruses and is a potent suppressor of host antiviral responses [1]. This suppression is mediated by the viral matrix (M) protein, which inhibits multiple steps in the expression of host genes [2], [3], [4], [5], [6], [7] including expression of genes that code for production of antiviral cytokines such as interferons [3], [8], [9]. M protein is a major structural component of the virus particle and plays several important roles in virus assembly [10]. However, the ability of M protein to suppress host gene expression is genetically separable from its function in virus assembly [3], [11].

M protein causes a global inhibition of host gene expression at multiple levels. M protein inhibits host transcription [2], [3], [4], [12], and inhibits nuclear-cytoplasmic RNA transport [6], [7], [13], [14] when expressed in transfected cells in the absence of other VSV components. M protein cannot inhibit host translation in the absence of other viral components [15]. However, in VSV-infected cells, host mRNA translation is inhibited and this inhibition is correlated with the ability of M protein to inhibit host transcription and transport [3], [5], [16].

One of the central questions in VSV pathogenesis is how does a relatively small (26 kDa) M protein cause such a profound inhibition of host gene expression? Since M protein lacks any enzymatic activity, it probably interferes with host gene expression by interacting with cellular proteins to alter their function. VSV M protein has been shown to interact with the host protein Rae1, which in turn interacts with the nucleoporin Nup98 [7], [14]. Rae1 had previously been thought to be involved in nuclear-cytoplasmic transport. Therefore, the global inhibition of host gene expression was attributed to a block in mRNA transport. However, other data show that Rae1 is not essential for nuclear-cytoplasmic mRNA transport, and silencing Rae1 expression does not inhibit cellular gene expression [17], [18]. Furthermore, a block in mRNA transport by M protein would not be consistent with earlier data showing that VSV inhibits host gene expression in mammalian cells primarily at the levels of transcription and translation rather than mRNA transport [2], [19], [20], [21]. With these discrepancies in mind, we decided to re-examine the interaction of M protein with Rae1 and Nup98 and the level of host gene expression in which they are involved.

Rae1 is localized in the cytoplasm and the nucleoplasm, as well as around the nuclear rim [18], [22], [23], [24]. Given its multiple sites of localization and the uncertainty about its function, we hypothesized that there may be multiple forms of Rae1 and that VSV M protein interacts with Rae1 to form multiple M protein-Rae1 complexes involved in inhibition of host gene expression. Indeed, we found that cellular Rae1 was present in high, intermediate, and low molecular weight complexes. The intermediate molecular weight complex with Nup98 was the form that interacted most effectively with M protein, but the low molecular weight form also interacted effectively with M protein in cells that overexpress Rae1 or cells in which expression of Nup98 was silenced. Silencing Rae1 expression did not affect host gene expression, but instead increased cellular resistance to the inhibitory effects of M protein. Furthermore, silencing Rae1 expression primarily affected the inhibition of host transcription, and had little if any effect on nuclear accumulation of host mRNA or translation of host proteins. These results support the idea that M protein-Rae1 complexes serve as platforms to promote the interaction of M protein with other factors involved in host transcription. They also support the idea that Rae1-Nup98 complexes play a previously under-appreciated role in regulation of cellular transcription.

## Results

### Specificity of interaction of M protein with Rae1 and Nup98

**Figure 1:**
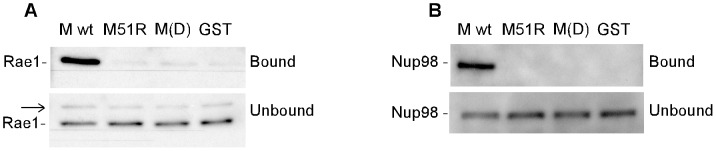
Specificity of interaction of M protein with Rae1 and Nup98. Recombinant wild type or mutant M protein GST fusion proteins or GST alone on glutathione beads were incubated for 1 hour with lysates from HEK 293 cells. Bound and unbound fractions were analyzed by immunoblotting and probed for Rae1 (**A**) or Nup98 (**B**). Arrow in unbound fraction in (**A**) indicates unrelated protein cross-reactive with Rae1 antibody (see Figure S1).

The purpose of the experiments in Figure 1 was to compare the ability to interact with Rae1 and Nup98 of wild type (wt) M protein to that of mutant M proteins that are defective in their ability to inhibit host gene expression. Viruses containing an arginine for methionine substitution at M protein amino acid 51 (M51R mutation) are fully functional in virus assembly [11], but are defective in inhibiting host transcription [3], [12], translation [16], and nuclear-cytoplasmic RNA transport [9]. Mutant M protein, M(D), which has three amino acid substitutions (D52A, T53A, and Y54A), has been shown to be defective in interacting with Rae1 and Nup98 [14]. This mutant M protein is defective in inhibiting host mRNA transport [7], [14], but has not been tested in its ability to inhibit host transcription and translation.

Recombinant wt or mutant M proteins were expressed in bacteria as fusion proteins with glutathione-S-transferase (GST) and purified on glutathione beads (Figure S1A). GST not fused to M protein was used as a negative control. Lysates from HEK 293 cells were incubated with recombinant GST-M proteins bound to glutathione beads, and bound fractions were analyzed by SDS-PAGE and immunoblotting using antibody against Rae1 (Figure 1A) or Nup98 (Figure 1B). Wt M protein interacted with Rae1 and Nup98, while no interaction was detected with M51R mutant M protein, similar to the negative controls. The unbound fractions also contained another band with slower electrophoretic mobility that was immunoreactive with the antibody against Rae1 (arrow in Figure 1A). Transfecting cells with Rae1 siRNA reduced Rae1 expression compared to control non-targeting siRNA, but had little if any effect on expression of the slower migrating band (Figure S1B), indicating that this band was not derived from Rae1.

Similar to endogenous Rae1, epitope-tagged Rae1 (HA-Rae1) expressed in transfected cells interacted with wt, but not mutant M protein (Figure S1C), and immunoprecipitation of HA-Rae1 from VSV-infected cells co-precipitated M protein (Figure S1D). Collectively, results in Figures 1 and S1 are in agreement with published reports that M protein interacts with Rae1 and Nup98 [7], [14]. In addition, the results with the M51R mutant M protein provide a genetic correlation between the interaction of M protein with these host proteins and the ability of recombinant viruses containing wt versus mutant M protein to inhibit host transcription and translation as well as nuclear-cytoplasmic transport. While the lack of interaction of the mutant M proteins with Rae1 correlates with their inactivity, these may not be causally related, as M protein could also interact with and inhibit other targets as well.

### Rae1 complexes capable of interacting with M protein

**Figure 2:**
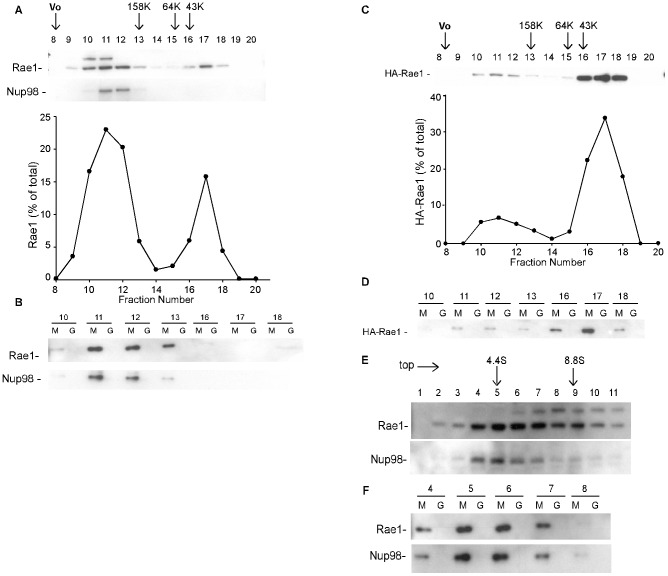
Gel filtration and sedimentation velocity analysis of complexes containing Rae1. (**A**) Cell lysates were chromatographed on a Superdex 200 column. Fractions were analyzed by immunoblots probed for Rae1 and Nup98. Arrows represent the fractions where standards of the indicated molecular weight eluted under the same conditions. The graph represents quantification of % Rae1 in each fraction normalized to total Rae1 eluting in all fractions. Vo indicates the void volume. (**B**) Column fractions from the same experiment as in (**A**) were incubated with wt GST-M protein (M) or GST (G) on glutathione beads for 1 hour. Bound fractions were analyzed by immunoblots probed for Rae1 and Nup98. (**C**) Cells were transfected with plasmid DNA encoding HA-Rae1. Lysates were chromatographed on a Superdex 200 column. Fractions were analyzed by immunoblots probed for HA. (**D**) Column fractions from (**C**) were incubated with wt GST-M protein (M) or GST (G) on glutathione beads for 1 hour. Bound fractions were analyzed by immunoblots probed for HA. (**E**) Cell lysates were subjected to sucrose gradient centrifugation. Fractions were collected from the top and probed for Rae1 (top panel) and Nup98 (bottom panel). Arrows represent fractions containing standards with the indicated s_20,w_ value subjected to the same conditions. (**F**) Sucrose gradient fractions from (**E**) were incubated with GST-M protein (M) or GST (G) on glutathione beads for 14 hours at 4°C. Bound fractions were analyzed by immunoblots probed for Rae1 and Nup98.

Rae1 interacts with multiple proteins involved in regulating mRNA transport [22], [25], [26], and in mitotic spindle [27] and checkpoint regulation [28]. To determine whether M protein interacts with Rae1 in different complexes, size exclusion chromatography was used to first separate complexes containing Rae1, then the Rae1 in these column fractions was tested for its ability to interact with M protein. In the experiment shown in Figure 2A, cell lysates were chromatographed on a Superdex 200 column, and fractions were analyzed using SDS-PAGE and immunoblotting with antibodies against Rae1 and Nup98. Rae1 was present in fractions 10–13 and in fractions 16–18, whereas Nup98 was present primarily in fractions 11–12. These data suggest that Rae1 exists in multiple forms: as high molecular weight complexes containing little if any Nup98 (corresponding to fraction 10), intermediate molecular weight complexes containing Nup98 (corresponding to fractions 11–12), and a low molecular weight form (corresponding to fractions 16–18). Approximately 30% of Rae1 was present in the low molecular weight form. The monomeric molecular weights of Nup98 and Rae1 are 98 and 42 kD, respectively. However, the low molecular weight form of Rae1 eluted in later fractions than would be expected compared to the ovalbumin standard which has a similar molecular weight. This is most likely due to the low molecular weight form of Rae1 adsorbing non-specifically to the chromatography matrix.

To determine which Rae1 complexes are competent to interact with M protein, the fractions containing Rae1 10–13 and 16–18 were tested for interaction with GST-M protein on glutathione beads. Shown in Figure 2B are immunoblots of the bound fractions obtained after incubation with GST-M protein or GST alone probed with antibodies against Rae1 or Nup98. Rae1 and Nup98 in fractions 11–13 were competent to interact with GST-M protein. However, the amount of Rae1 in fraction 10 that interacted with GST-M protein was much less than that in fractions 11–13, indicating that the high molecular weight complex was relatively ineffective in interacting with M protein, compared to the intermediate molecular weight complexes (corresponding to fractions 11–13). Very little Rae1 in fractions 16–18 interacted with GST-M protein. However, the interaction was detectable on longer exposures (data not shown).

To determine whether overexpression of epitope-tagged Rae1 alters the complexes competent to interact with M protein, HEK293 cells were transfected with plasmid DNA encoding HA-Rae1 as described [14], and cell lysates were analyzed by gel filtration and immunoblotting with anti-HA antibody. As shown in Figure 2C, HA-Rae1 was present in fractions 10–13 and 16–18, similar to endogenous Rae1. However, most of the HA-Rae1 was in the low molecular weight fractions (approximately 80%). HA-Rae1 in the low molecular weight fractions (16–18) was competent to interact with GST-M protein (Figure 2D), as was the HA-Rae1 in the intermediate molecular weight fractions (11–13). The interaction of GST-M protein with Rae1 or HA-Rae1 was not affected by post-translational modifications that alter the charge of Rae1, as shown by isoelectric focusing of bound and unbound fractions followed by immunoblotting for Rae1 or HA-Rae1 (Figure S2). Collectively, the data in Fig. 2A–D indicate that M protein can interact with Rae1 in the low molecular weight form as well as the intermediate molecular weight complex with Nup98.

Cell lysates were subjected to rate zonal centrifugation using sucrose gradients to further estimate the sizes of complexes containing Rae1. After centrifugation twenty fractions were collected and analyzed by SDS-PAGE and immunoblotting. Shown in Figure 2E are fractions 1–11 collected from the top of the gradient. Rae1 was present in a broad peak from fractions 3–9, with a peak in fractions 5–6, which corresponds to an s_20.w_ value of 5±1S (average ± SD for 5 experiments). This peak was composed primarily of the Rae1 complexes containing Nup98, which interacted effectively with GST-M protein (Figure 2F), whereas GST-M protein did not interact efficiently with Rae1 in fraction 8 of the sucrose gradients which had much less Nup98. Rae1-Nup98 complexes were not well-resolved from the low molecular weight form of Rae1 by sedimentation analysis. The observation that the Rae1-Nup98 complexes eluted close to the high molecular weight complexes in gel filtration but close to the low molecular weight form in sedimentation is typical of proteins with a larger Stokes radius than similarly sized compactly folded globular proteins, suggesting that these complexes have either elongated structures or intrinsically disordered regions (see Discussion).

### Impact of silencing Rae1 or Nup98 expression on complexes that interact with M protein

**Figure 3:**
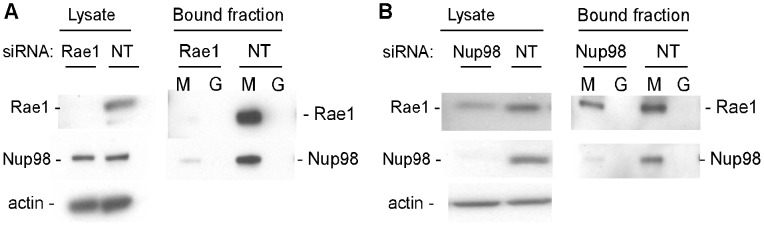
Effects of silencing the expression of Rae1 and Nup98 on interaction with M protein. (**A**) HeLa cells were transfected with Rae1 siRNA or with non-targeting (NT) siRNA. 48 hours post-transfection, lysates from Rae1 siRNA cells or NT siRNA cells were incubated with GST-M protein (M) or GST (G) on glutathione beads for 2 hours at 4 C. Cell lysates and bound fractions were analyzed by immunoblots probed for Rae1 or Nup98. (**B**) Cells were transfected with Nup98 siRNA or with non-targeting (NT) siRNA and analyzed as in (**A**).

The impact of silencing expression of Rae1 or Nup98 on complexes that interact with M protein was determined by transfecting HeLa cells with siRNAs specific for each mRNA or nontargeting (NT) control siRNA. By selecting the most effective among four Rae1 siRNAs and four different transfection reagents, Rae1 expression levels were reduced to less than 2% of those in NT siRNA controls, but had little if any effect on expression of Nup98 (Figure 3A). Silencing the expression of Nup98 (Figure 3B) reduced Nup98 protein levels to 10% of those in NT siRNA controls. There was a slightly lower level of expression of Rae1 in lysates from Nup98 siRNA cells (70±9% of NT siRNA cells, as determined by densitometry of 5 separate experiments). As reported previously [26], silencing the expression of Rae1 was not lethal to the cells, and silencing the expression of Nup98 also had little if any effect on cell viability in the time period of these experiments (48–72 hours post-transfection). In the experiments shown in Figure 3, cell extracts were prepared 48 hours post-transfection and were incubated with GST-M protein or GST alone on glutathione beads. Cell extracts and the bound fractions were analyzed by immunoblotting with antibodies against Rae1 and Nup98. In lysates from Rae1 siRNA cells, the amount of Nup98 that interacted with M protein was considerably less than that in lysates from NT siRNA cells (Figure 3A). Quantification of immunoblots from multiple experiments indicated that the amounts of Nup98 that interacted with M protein in lysates from Rae1 siRNA cells were <10% of those in lysates from NT siRNA cells. These results are consistent with previous mutagenesis experiments [14] indicating that Rae1 is required for the interaction of M protein with Nup98.

**Figure 4:**
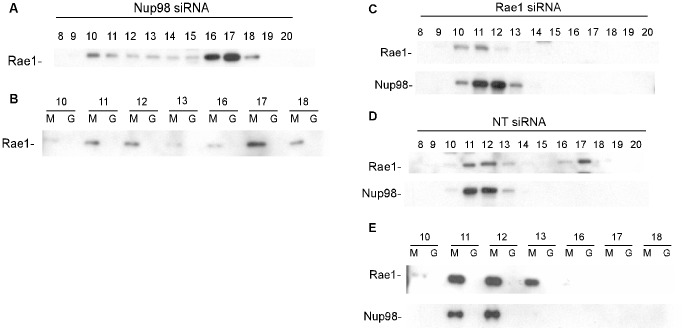
Effects of silencing the expression of Nup98 or Rae1 on complexes containing Rae1. HeLa cells were transfected with Nup98 siRNA (**A**), Rae1 siRNA (**C**), or non-targeting siRNA (**D**). 48 hours post-transfection, lysates were chromatographed on a Superdex 200 column. Fractions were analyzed by immunoblots probed for the indicated proteins. (**B**) and (**E**): Column fractions from (**A**) and (**D**), respectively, were incubated with GST-M protein (M) or GST (G) on glutathione beads for 1 hour, and bound fractions were analyzed by immunoblots probed for Rae1 and Nup98.

The amount of Rae1 in lysates from Nup98 siRNA cells that interacted with M protein was reduced slightly (Figure 3B), but this could be attributed to the lower levels of Rae1 expression. These results suggest that Nup98 is not required for Rae1 to interact with M protein, although it is also possible that residual Nup98 expression in Nup98 siRNA cells could mediate the interaction. To distinguish these possibilities, and to determine whether silencing of Nup98 expression affected the distribution of Rae1 complexes, the elution profile of Rae1 in cells transfected with Nup98 siRNA was determined (Figure 4A). In Nup98 siRNA cells, Rae1 eluted primarily in fractions corresponding to the low molecular weight form (16–18). When the fractions containing Rae1 were incubated with GST-M protein, the low molecular weight form of Rae1 was competent to interact with M protein, as were the residual intermediate molecular weight complexes containing Rae1 and Nup98 (Figure 4B). These data indicate that with the low levels of Nup98 expression, Rae1 primarily exists in a low molecular weight form that is competent to interact with M protein.

In Rae1 siRNA cells, Rae1 could not be detected in the intermediate or low molecular weight forms. Following concentration of cell lysates, the residual Rae1 could be detected primarily in the high molecular weight form (Figure 4C, fractions 10–11). There was little if any effect of silencing Rae1 expression on elution of Nup98, which was similar to that in NT siRNA cells (Figure 4D). Similar to the data in Figure 2, in siNT cells the intermediate molecular weight Rae1 complexes containing Nup98 bound efficiently to M protein (Figure 4E).

### Rae1 is required for VSV to inhibit host transcription

**Figure 5:**
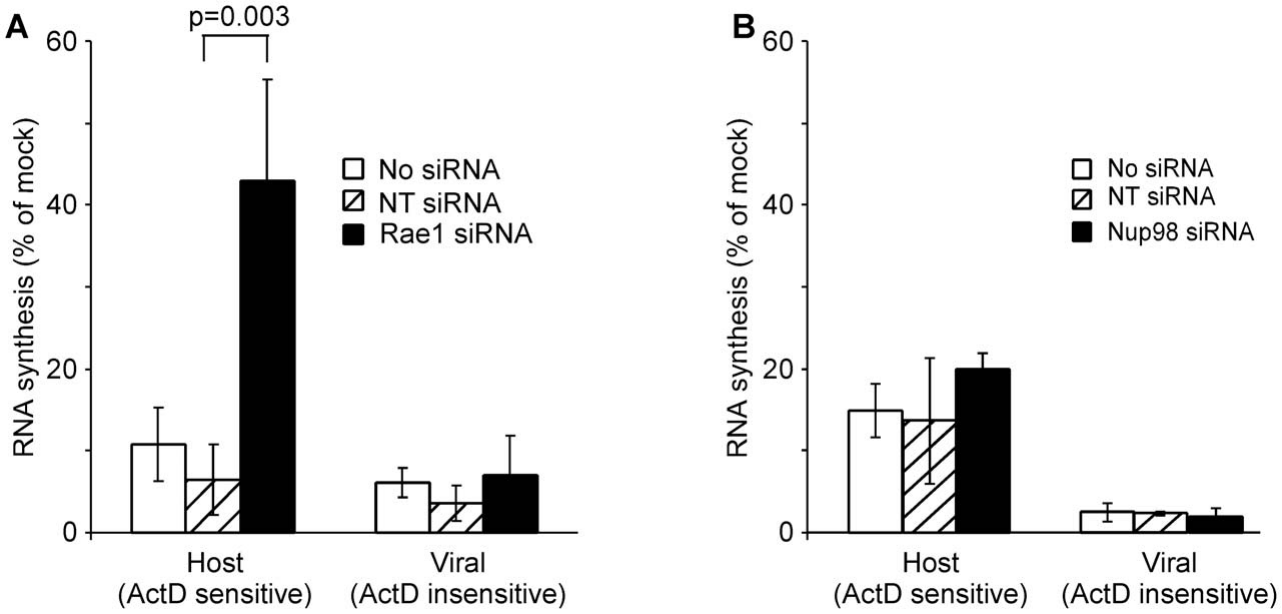
Effects of silencing the expression of Rae1 or Nup98 on host and viral transcription in VSV-infected cells. HeLa cells were either not transfected or transfected with Rae1 siRNA (**A**), Nup98 siRNA (**B**) or non-targeting (NT) siRNA. At 72 hours post-transfection, cells were either mock or infected with recombinant wild-type (rwt) virus for 6 hours in the presence or absence of actinomycin D (ActD, 5 µg/ml). Cells were labeled with [^3^H] uridine for 30 minutes. Cells were lysed and RNA was precipitated using trichloroacetic acid, and acid precipitable radioactivity was measured. The graph represents host (ActD sensitive) and viral (ActD insensitive) RNA synthesis expressed as a percentage of total RNA synthesis in mock infected cells as illustrated in Table 1. The data shown are means ± standard deviation from five independent experiments.

The effects of silencing Rae1 and Nup98 on VSV's ability to inhibit host transcription were determined. Host transcription was quantified by incorporation of [^3^H] uridine into RNA. This approach measures RNA synthesis by all three host RNA polymerases, with RNA polymerase I making the largest contribution. HeLa cells were transfected with siRNA against Rae1 or Nup98 or with NT siRNA. At 72 hours post-transfection, cells were mock infected or infected with recombinant wild-type (rwt) virus in the presence or absence of actinomycin D, an inhibitor of host transcription, which does not affect transcription by the viral RNA-dependent RNA polymerase. At 6 hours postinfection, cells were pulse labeled with [^3^H] uridine, and cell lysates were analyzed for trichloroacetic acid-precipitable radioactivity. Data from a representative experiment for Rae1 and Nup98 siRNA is shown in Table 1A and B, respectively, and results of multiple experiments are summarized in Figure 5A and B. In virus-infected cells, RNA synthesis in the absence of actinomycin D represents synthesis of both host RNA and viral RNA. Synthesis in the presence of actinomycin D represents viral RNA synthesis. Thus host RNA synthesis was determined by subtracting the amount of RNA synthesized in the presence of actinomycin D from the amount in the absence of actinomycin D. Both host (actinomycin D sensitive) and viral (actinomycin D resistant) RNA synthesis were expressed as a percentage of total RNA synthesis in mock-infected controls in order to normalize data among multiple experiments.

**Table 1:** Effect of silencing Rae1 or Nup98 expression on RNA synthesis in VSV-infected cells.

A. Rae1		^3^H cpm (×10^−3^) ± s.d.			% of mock	
siRNA	Infection	−Act D^a^	+Act D^a^	Difference	Host	Viral
None	Mock	54.1±19.4	0	54.1	100	-
	VSV	9.4±0.2	2.4±0.8	7.0	12.9	4.4
NT^a^	Mock	96.2±48.1	0	96.2	100	-
	VSV	4.7±1.7	1.5±0.2	3.2	3.3	1.6
Rae1	Mock	50.8±8.5	0	50.8	100	-
	VSV	28.9±14.3	2.6±0.7	26.3	51.8	5.1

B. Nup98		^3^H cpm (×10^−3^) ± s.d.			% of mock	
siRNA	Infection	−Act D^a^	+Act D^a^	Difference	Host	Viral
None	Mock	108.6±57.4	0	108.6	100	-
	VSV	22.7±2.4	3.4±1.2	19.3	17.7	3.15
NT^a^	Mock	143.1±49.1	0	143.1	100	-
	VSV	29.9±6.0	3.6±2.5	26.3	18.3	2.52
Nup98	Mock	69.7±32.0	0.08±0.06	69.0	99.0	-
	VSV	15.0±2.8	2.1±0.1	12.9	18.6	3.0

HeLa cells transfected with the indicated siRNA were either mock-infected or infected with VSV in the presence or absence of actinomycin D. At 6 h postinfection, cells were labeled with ^3^H uridine for 30 min; RNA was acid-precipitated, and radioactivity was determined by scintillation counting. Data shown are mean ± s.d. for triplicate cultures from a representative experiment.

a Abbreviations: ActD - actinomycin D; NT – non-targeting siRNA.

Host RNA synthesis in NT siRNA cells or non-transfected control cells infected with rwt virus was reduced to approximately 10% of that in mock-infected cells. However, host RNA synthesis in Rae1 siRNA cells infected with rwt virus continued at approximately 40% of that in mock-infected cells. Viral RNA synthesis was similar in all three cell types. These results indicate that cells transfected with Rae1 siRNA are more resistant to inhibition of host transcription by VSV, and thus the expression of Rae1 is important for the ability of VSV to inhibit host transcription. In contrast to Rae1, silencing the expression of Nup98 had no significant effect on host transcription inhibition by VSV (Figure 5B).

### Silencing Rae1expression affects the expression of RNA polymerase II-transcribed mRNAs

Labeling with ^3^H-uridine (Figure 5) measures RNA synthesis by all three host RNA polymerases. RNA polymerases I, II, and III, have similar sensitivities to the inhibitory activity of M protein [2], [19], [20], [21], so it was expected that RNA polymerase II-dependent transcription would be affected by silencing Rae1 to an extent similar to total RNA synthesis. To specifically test the effect of silencing Rae1 expression on RNA polymerase II transcripts, host mRNAs were analyzed by cDNA microarrays. If the principal effect of M protein is to inhibit transcription, synthesis of mRNAs encoding antiviral proteins that would otherwise be induced by virus infection will be prevented, and the levels of mRNAs that are rapidly turned over will be reduced. Conversely, if silencing Rae1 expression increases cellular resistance to inhibition of transcription by M protein, mRNAs characteristic of host antiviral responses should be increased, and mRNAs that are rapidly turned over should be reduced to a lesser extent than in cells transfected with control siRNA.

**Figure 6:**
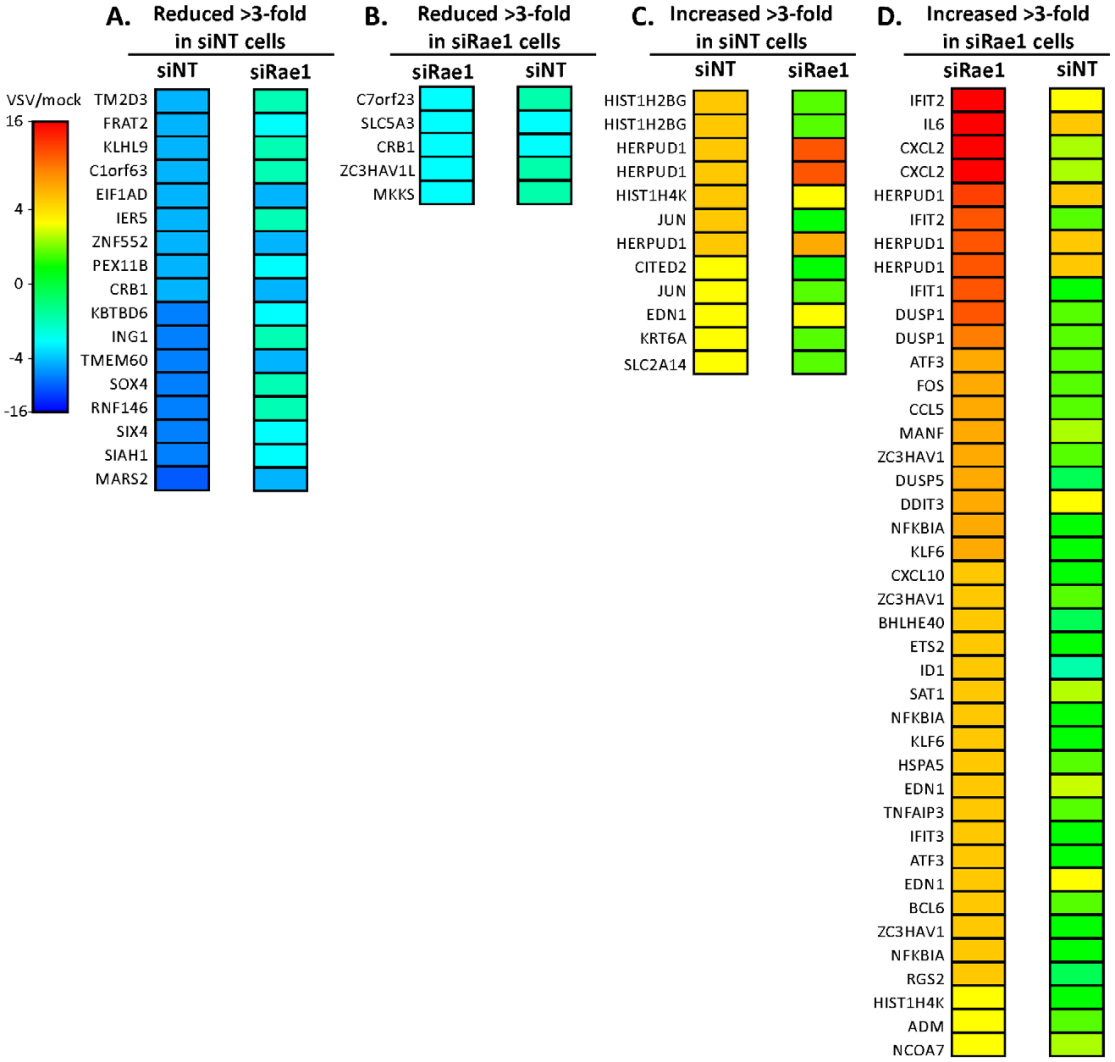
Effects of silencing the expression of Rae1 on mRNA expression in VSV-infected cells. Cells were transfected with Rae1 siRNA or NT siRNA, then mock-infected or infected with rwt virus. At 6 h postinfection, total RNA was isolated and analyzed using Affymetrix Human Genome U219 Array strips. Data shown are gene symbols of probe sets that were reproducibly decreased (**A** and **B**) or increased (**C** and **D**) by greater than 3-fold in VSV-infected versus mock-infected siNT cells (**A** and **C**) or siRae1 cells (**B** and **D**). The selection criteria were that the combined variance in the probe set in repeat experiments gave p<0.005 to minimize the false discovery rate. The probe sets, definition of gene symbols, and numerical data are provided in Table S1.

Figure 6 and Table S1 show the probe sets for genes that were reproducibly increased or decreased by greater than 3-fold at 6 hr postinfection with VSV compared to mock infection of siNT cells and siRae1 cells. To minimize the false discovery rate, the selection criteria for Figure 6 and Table S1 were that the pooled variance in the probe set in repeat experiments gave p<0.005 in comparing VSV-infected to mock-infected cells. In siNT cells, 880 probe sets met this criterion, most of which were only slightly different. The geometric mean intensity ratio (VSV/mock) of all the probe sets that met the selection criteria was 0.849, indicating that the primary effect of VSV infection was a reduction in mRNA levels. In siRae1 cells, 970 probe sets met the selection criterion, with a geometric mean intensity ratio of 1.016, which was significantly different from that of siNT cells (p<10^−12^ by Students t-test). The selection of a 3-fold difference as a criterion for Figure 6 and Table S1 was based on a comparison of mock-infected siRae1 versus mock-infected siNT cells. In this comparison, 713 probe sets had p<0.005. Of these only 3 were decreased >3-fold (Table S1 E), including Rae1 itself. Thus, using a 3-fold difference as a selection criterion focused attention on gene expression changes in response to virus infection that were greater than the effects of silencing Rae1 expression in mock-infected cells. However, the same conclusions could be drawn by using a 2-fold increase or decrease as a selection criterion (not shown).

Few host mRNAs were decreased by more than 3-fold during the 6 hr timecourse of VSV infection, since the typical half-life of HeLa cell mRNAs is 6–12 hr [29]. However, those that were reduced >3-fold in siNT cells were reduced to a much lesser extent in siRae1 cells (Figure 6A). Also fewer genes were reduced >3-fold in siRae1 cells (Figure 6B) compared to siNT cells (Figure 6A). Infection with VSV increased expression of relatively few genes >3-fold in siNT cells (Figure 6C), which is consistent with the inhibition of host transcription. The ones that were induced include stress-induced mRNAs such as those encoding c-Jun and homocysteine-inducible, endoplasmic reticulum stress-inducible, ubiquitin-like domain member 1 (HERPUD1). In siRae1 cells, many more genes were induced >3-fold than in siNT cells (Figure 6D). The striking result is that many of these genes encode cytokines and other antiviral proteins that are typically induced by virus infection, such as IL-6, CXCL2, CCL5, CXCL10, and IFIT1, 2 and 3. The results with c-Jun and IL-6 were confirmed by real-time RT-PCR. As an additional control, silencing Rae1 expression had no effect on induction of IL-6 mRNA in cells infected with an M protein mutant virus (rM51R-M virus [3], [30]) as determined by real-time RT-PCR (not shown). Collectively, the results in Figure 6 are fully consistent with the conclusion that silencing Rae1 expression increases cellular resistance to the inhibitory effects of M protein at the level of host transcription. Furthermore, they emphasize the importance of the M protein-Rae1 complex in suppressing the antiviral response of host cells.

### Silencing Rae1expression has little effect on nuclear accumulation of host mRNA

**Figure 7:**
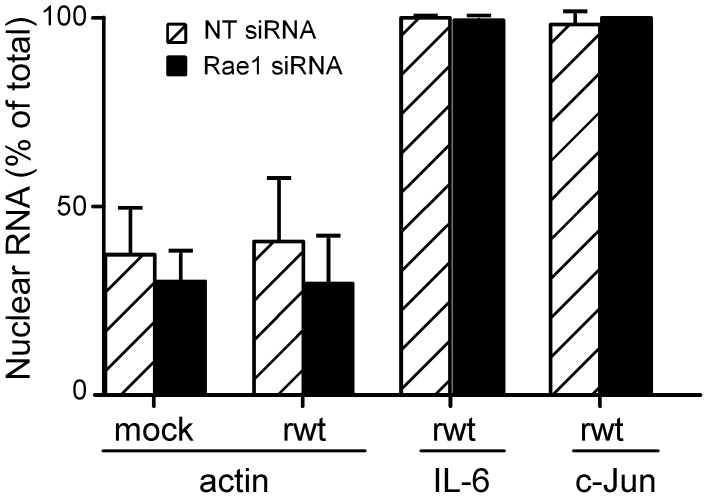
Effects of silencing the expression of Rae1 on accumulation of host mRNAs in nuclei of VSV-infected cells. Cells were transfected with Rae1 siRNA or NT siRNA and were either mock infected or infected with rwt virus for 6 hours. Cells were lysed and separated into nuclear and cytoplasmic fractions. *E.coli* mRNA was added to each fraction to quantify recovery of RNA. Actin, IL-6, or c-Jun transcripts were measured in each fraction using real time RT PCR and normalized to the amount of *E.coli* uidA transcripts. The graph represents the amount of nuclear mRNA expressed as a percentage of total mRNA. The data shown are means ± standard deviation from three independent experiments for actin and two independent experiments for IL-6 and c-Jun.

To determine the effect of silencing Rae1 expression on the accumulation of host mRNA in the nucleus, the amount of host mRNAs in the nucleus versus cytoplasm was measured by real time RT-PCR in cells transfected with siRNA against Rae1 or with NT siRNA. At 72 hours post-transfection, cells were mock infected or infected with rwt virus. At 6 hours postinfection, cells were lysed and separated into nuclear and cytoplasmic fractions, and RNA was isolated from each fraction. The nuclear fractions were largely free of cytoplasmic contamination, since 28S and 18S rRNAs were undetectable by gel electrophoresis and ethidium bromide staining (data not shown). As a control to monitor the efficiency of RNA recovery in the nuclear and cytoplasmic fractions, a known quantity of *E.coli* mRNA was added to the cytoplasmic and the nuclear fractions before harvesting RNA. The amounts of host mRNAs measured by real time RT-PCR in the cytoplasmic and nuclear fractions were normalized to the amount of *E.coli* uidA transcript measured in the same samples, and the percentage of total mRNA in the nucleus was calculated (Figure 7). Three different host mRNAs were analyzed. Actin mRNA was assayed as a representative mRNA for housekeeping genes, and IL-6 and c-Jun mRNAs were assayed as representative mRNAs induced during VSV infection.

The amounts of actin mRNA in nuclei of mock-infected Rae1 siRNA cells and NT siRNA cells were similar, approximately 40% of total actin mRNA, similar to previous data for this cell type [31], [32]. This result is consistent with the idea that Rae1 is not required for transport of actin mRNA. There was no significant difference in the levels of actin mRNA in the nuclei of rwt virus-infected cells compared to mock-infected cells for either Rae1 siRNA or NT siRNA cells. This result is consistent with earlier data indicating that there is little if any net accumulation of housekeeping mRNAs in the nuclei of VSV-infected cells, due to the inhibition of host transcription [20], [21]. However, nearly all (>99%) of the IL-6 and c-Jun mRNA was in the nuclear fraction following infection with rwt virus. There was no significant difference between Rae1 siRNA cells versus NT siRNA cells in the nuclear accumulation of any of the host mRNAs assayed following virus infection. Collectively these results indicate that silencing Rae1 expression has little if any effect on the nuclear accumulation of host mRNA in VSV-infected cells.

### Silencing Rae1 expression does not affect VSV's ability to inhibit host translation

**Figure 8:**
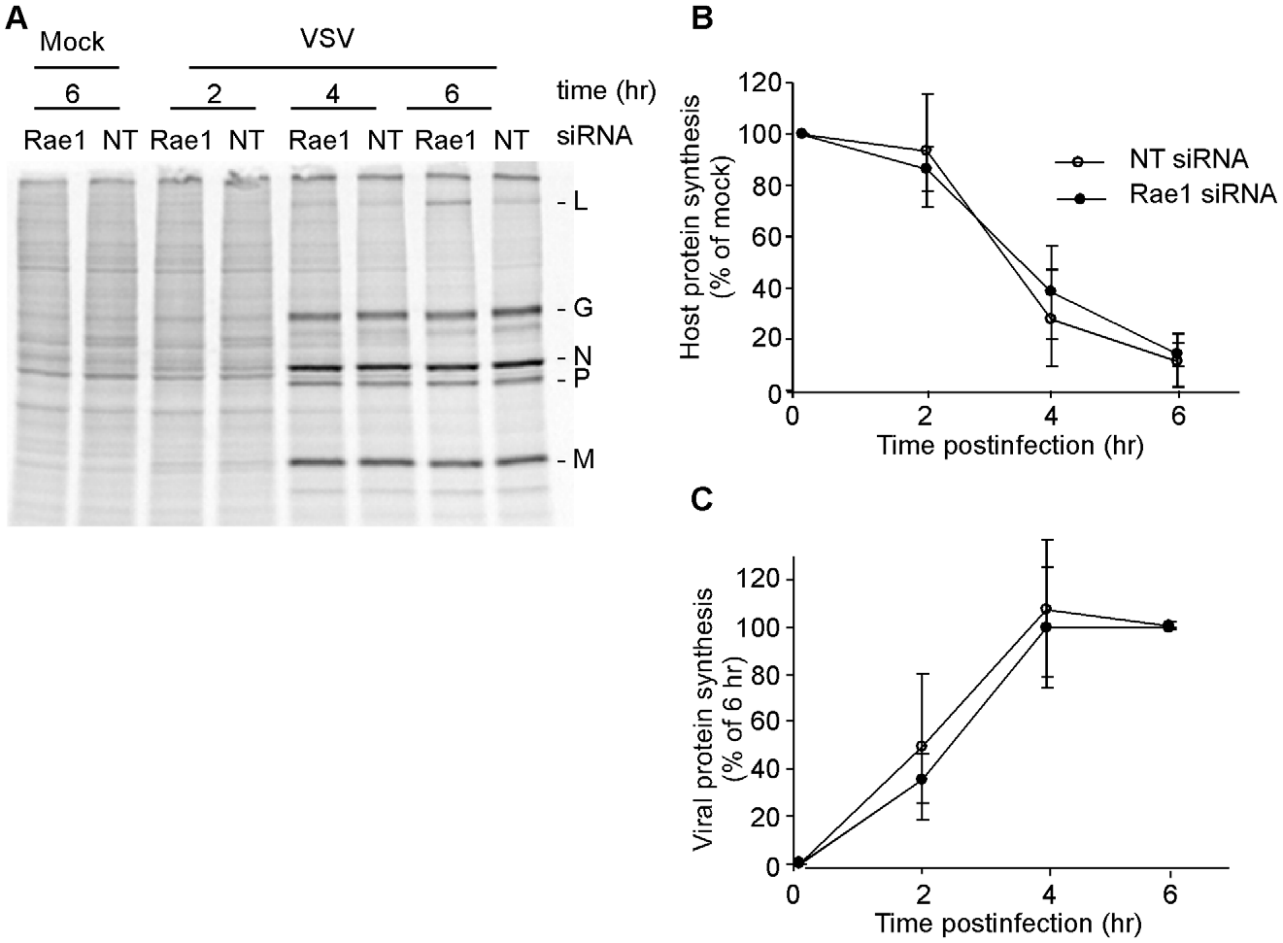
Effects of silencing the expression of Rae1 on host and viral protein synthesis. (**A**) HeLa cells were transfected with Rae1 siRNA or non-targeting (NT) siRNA. 72 hours post-transfection, cells were mock infected or infected with rwt virus for the indicated times and labeled with [^35^S ] methionine for 10 min. Lysates from Rae1 siRNA cells or NT siRNA cells were analyzed by SDS-PAGE and phosphoimaging. Shown is a phosphoimage with the viral proteins indicated on the right. (**B**) Quantification of host protein synthesis expressed as a percentage of mock infected cells. Data shown are the means ± standard deviation of three separate experiments. (**C**) Quantification of viral protein synthesis expressed as a percentage of synthesis at six hours postinfection. Data shown are the means ± standard deviation of three separate experiments.

To determine the effects of silencing the expression of Rae1 on the ability of VSV to inhibit host translation, cells were transfected with Rae1 siRNA or with NT siRNA for 72 hours, and the rates of host and viral protein synthesis were determined at varying times postinfection. Cells were pulse-labeled with [^35^S] methionine, and lysates were analyzed by SDS-PAGE and phosphorescence imaging. Figure 8A shows a phosphoimage that compares protein synthesis in Rae1 siRNA cells versus NT siRNA cells that were mock infected or infected with rwt virus for 2, 4, and 6 hours. The ladder of bands in mock infected cells represents synthesis of host proteins. Host protein synthesis was quantified from regions of the gel devoid of viral proteins in three separate experiments and is shown in Figure 8B expressed as a percentage of that in mock-infected cells. Viral protein synthesis was quantified from radioactivity in all the viral bands and is expressed as a percent of synthesis at 6 hours postinfection (Figure 8C). During infection with rwt virus, the synthesis of host proteins was inhibited in both Rae1 siRNA and NT siRNA cells, and was almost completely inhibited at 6 hours post infection. In rwt virus-infected cells, the synthesis of viral proteins L, G, N, P and M can be observed at 2 hours postinfection and by 6 hours the synthesis has reached its maximum [5]. There was no significant difference between Rae1 siRNA cells and NT siRNA cells in either the inhibition of host protein synthesis or the levels of viral protein synthesis. These data indicate that silencing expression of Rae1 does not affect the ability of VSV to inhibit host translation. Similarly, silencing expression of Nup98 did not affect the ability of VSV to inhibit host translation or affect viral protein synthesis (Figure S3).

### Cells transfected with Rae1 siRNA are more resistant to inhibition of host gene expression by M protein expressed in the absence of other VSV components

The results shown above indicate that Rae1 is required for efficient inhibition of host transcription in VSV- infected cells, but has little if any effect on nuclear accumulation of host mRNA or the shut-off of host translation. To further address whether M protein's interaction with Rae1 is required for M-protein-induced inhibition of host gene expression, M protein's ability to inhibit gene expression in transfected cells in the absence of other viral components was measured in cells silenced for the expression of Rae1.

Host-directed gene expression in transfected cells was measured using a luciferase reporter driven by the SV40 early promoter, which is dependent on the host transcriptional apparatus. Rae1 siRNA cells, NT siRNA cells, or cells that received no siRNA were cotransfected with luciferase plasmid DNA together with varying amounts of *in vitro*-transcribed mRNA encoding M protein or control RNA. The cells were transfected with M mRNA rather than plasmid DNA encoding M protein to promote optimal expression of M protein, because M protein inhibits transcription of its own mRNA from plasmid DNA that depends on host transcription machinery [4], [15]. At 24 hours post-transfection cells were lysed and luciferase activity was measured.

**Figure 9:**
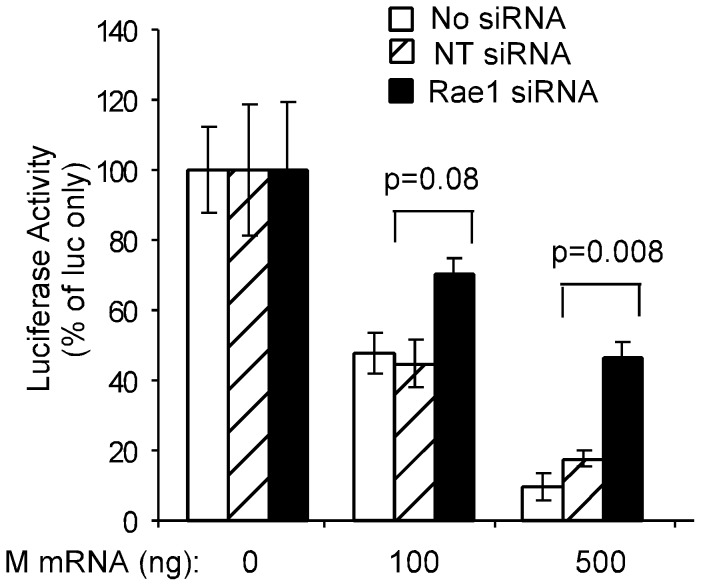
Effect of silencing Rae1 expression on the inhibition of host gene expression by M protein expressed in the absence of other VSV components. HeLa cells were either not transfected or transfected with Rae1 siRNA or non-targeting (NT) siRNA. After 48 hours, cells were re-seeded, then cotransfected with plasmid DNA encoding luciferase under the control of the SV40 promoter together with the indicated amounts of M mRNA. The amount of total RNA was kept constant by addition of yeast tRNA. At 24 hours post-transfection, cells were lysed and luciferase reporter activity was measured. The graph represents activity expressed as a percentage of average luciferase activity in the absence of M mRNA. Data shown are means ± standard deviation from three independent experiments.

Shown in Figure 9 is luciferase activity that was normalized to the activity in the absence of M mRNA. In control cells transfected with NT siRNA or no siRNA, expression of M protein inhibited luciferase expression in a dose-dependent manner to approximately 10% of control with 500 ng of M mRNA, similar to previous results [15], [33]. However, in Rae1 siRNA cells, there was significantly higher luciferase expression at both doses of M mRNA, with luciferase activity approximately 50% of control in cells transfected with 500 ng of M mRNA. This result is consistent with the results in Figures 5 and 6 that silencing the expression of Rae1 increases the resistance of cells to M protein's inhibitory effects on host gene expression.

### Rae1-Nup98 complexes associated with chromatin fractions in the nucleus are competent to interact with M protein

**Figure 10:**
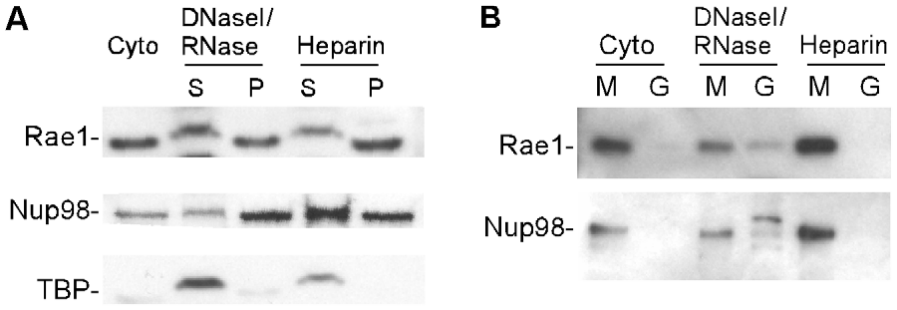
Interaction of M protein with Rae1 and Nup98 in nuclear chromatin-associated fractions. HeLa cells were lysed in hypotonic buffer and separated into nuclei and cytoplasm (Cyto). Nuclei were fractionated as described in [36]. (**A**) Supernatant (S) and pellet (P) fractions were analyzed by immunoblots probed for the presence of Rae1, Nup98, and TATA-binding protein (TBP). (**B**) Supernatant fractions from (**A**) were incubated with GST-M protein (M) or GST (G) on glutathione beads. Bound fractions were analyzed by immunoblots probed for Rae1 and Nup98.

It has recently been shown that Nup98 plays a role in regulation of transcription and is associated with transcriptionally active chromatin in the nucleoplasm as well as nuclear pores [34], [35]. The observation that silencing Rae1 expression primarily affects the ability of VSV to inhibit host transcription suggests that M protein binds to Rae1-Nup98 complexes associated with chromatin in the nucleus. This hypothesis was tested by isolating chromatin-associated Rae1-Nup98 complexes from nuclei of HeLa cells lysed in hypotonic buffer and fractionated as described in [36]. Nuclei were treated with DNase and RNase followed by heparin, a negatively charged polyanion, to solubilize chromatin and chromatin-associated proteins. The pellet largely represents the nuclear envelope. These fractions were probed for the presence of Rae1 and Nup98 and for their ability to interact with GST-M protein (Figure 10).

The cytoplasmic fraction as well as the pellet and supernatant fractions obtained after each treatment of the nuclei were analyzed by SDS-PAGE and immunoblotting with antibodies against Rae1 and Nup98 (Figure 10A). As a control to monitor the effectiveness of the solubilization of chromatin-associated proteins, the fractions were also probed for the transcription factor TATA-binding protein (TBP). Rae1 and Nup98 were present in the cytoplasmic fraction as well as the supernatant fractions after treatment with DNase/RNase and heparin that contain proteins associated with chromatin, as expected from previous data [7], [18], [22], [23], [24], [37], [38]. The fact that both Rae1 and Nup98 are in the chromatin-containing fractions does not show that they function in concert in that environment. However, they do appear to be present as a complex that is competent to bind M protein, since GST-M protein co-precipitated Rae1 and Nup98 from both the cytoplasmic fraction and nuclear fractions containing chromatin-associated proteins (Figure 10B). These data indicate that M protein can interact with Rae1-Nup98 complexes in these compartments, and are consistent with the idea that Rae1-Nup98 complexes are involved in the inhibition of transcription in VSV-infected cells.

## Discussion

One of the remarkable aspects of VSV pathogenesis is the ability of M protein to induce pleiotropic effects in infected cells. M protein plays multiple roles in both virus assembly and in the inhibition of host gene expression [10]. M protein inhibits transcription by all three host RNA polymerases [2], [3], [4], [12], inhibits nucleo-cytoplasmic RNA transport [6], [7], [13], and plays a role in inhibition of translation of host mRNA [3], [5], [16]. One of the mechanisms by which M protein may serve these diverse functions is through interaction with host proteins, such as Rae1, that may also serve multiple functions in a cell.

Previous data had shown that M protein interacts with Rae1-Nup98 complexes, but did not address the ability of M protein to interact with other forms of Rae1. It was originally thought that interaction of M protein with Rae1-Nup98 complexes was responsible for blocking nuclear-cytoplasmic transport. Therefore our hypothesis was that M protein interacts with other forms of Rae1 to inhibit other steps in host gene expression, such as transcription and translation. However, the data presented here show that Rae1-Nup98 complexes are the major form of Rae1 capable of interacting with M protein. In situations where the cellular levels of Rae1 or Nup98 are altered, either by overexpressing Rae1 or silencing expression of Nup98, the low molecular weight form of Rae1 also interacts with M protein. Furthermore, rather than affecting the accumulation of host RNA in the nucleus, the major effect of silencing Rae1 expression was to make the cells more resistant to the inhibition of transcription by VSV. These results lead to a new model for how the interaction of M protein with Rae1 inhibits host gene expression. They also support the idea that Rae1-Nup98 complexes play a previously under-appreciated role in regulation of cellular transcription.

M protein does not inhibit host gene expression simply by interfering with Rae1 function, since Rae1 is not essential for host gene expression [17], [18]. This raises the question of how interaction of M protein with a sub-population of a protein that is not essential for gene expression can have a global effect on host gene expression at multiple levels. To address this paradox, we propose a model where M protein interacts with Rae1-Nup98 complexes that serve as a platform for M protein to interact with other essential host proteins, thereby, interfering with their function. The “platform hypothesis” predicts the opposite effects of silencing Rae1 expression compared to hypotheses based on M protein inhibition of Rae1 function. The latter hypotheses predict that Rae1 siRNA cells should be more sensitive than control cells to the effects of M protein, because of the lower level of Rae1 expression. In contrast the “platform hypothesis” predicts that Rae1 siRNA cells should be less sensitive to the effects of M protein than controls, since there is less Rae1 to mediate the interaction of M protein with other targets. Our data showing that Rae1 siRNA cells are relatively resistant to the inhibitory effects of M protein (Figures 5, 6, and 9) provide support for the platform hypothesis and are largely inconsistent with hypotheses based on M protein interference with Rae1 function.

The structural features of Rae1-Nup98 complexes are well-suited to mediate the interaction of M protein with other cellular targets. Rae1 is a member of the family of WD repeat proteins [22], [23], [24], which are known to adopt beta propeller folds [39], [40], [41] that have large surface areas suitable for multiple protein interactions. Human Rae1, which has four WD repeats in its sequence [22], [24], has been shown to form seven bladed β propellers with extensive surface loops [42], which provide large surface areas that could serve as interacting regions for M protein to disrupt function of other proteins associated with Rae1. Nup98 has a small globular region near its C-terminus. However, most of the remaining sequence contains FG-repeats, which are intrinsically disordered in other FG-repeat-containing proteins [43]. The FG-repeat region of Nup98 provides sites of interaction with a wide variety of other cellular proteins [43]. Rae1-Nup98 complexes have a larger apparent Stokes radius in gel filtration and a smaller sedimentation velocity than would be expected of compactly folded proteins. This could be due to an elongated structure, but is most likely due to the presence of disordered regions in the protein sequence. From our gel filtration data and that of Matsuoka *et al* [44], the Stokes radius of the Rae1-Nup98 complex is estimated to be 70–75 Å, which combined with an estimated s_20,w_ of 5S (Figure 2) gives a molecular weight of approximately 150,000. This is a reasonable result for a 1∶1 complex of Rae1 and Nup98.

Our data also support the idea that the interaction of M protein with Rae1-Nup98 complexes inhibits host gene expression by inhibiting host transcription (Figure 5). Previous data had suggested that Rae1 and Nup98 interact with the transcriptional machinery. Both Rae1 and Nup98 are present in the nucleoplasm, as well as the nuclear envelope and cytoplasm [7], [18], [22], [23], [24], [37], [38]. The localization of Rae1 at the nuclear envelope is affected by inhibitors of RNA polymerase I and II activity [22]. This suggests that the localization of Rae1 is dependent on ongoing transcription. Similarly, the mobility of Nup98 in the nucleus is also dependent on ongoing transcription [37]. Nup98 in the nucleoplasm has been shown to interact with developmentally regulated genes in *Drosophila*, and altering Nup98 expression alters the expression of these genes, implicating Nup98 in the control of transcription [34], [35].

Recent evidence suggests that the steps involved in gene transcription, nascent mRNA processing, and transport are coupled [45], [46]. Rae1 can be cross-linked to poly A-containing mRNA [23], and Rae1 interacts with other mRNA binding proteins [26], suggesting that Rae1 and Nup98 may be a part of larger ribonucleoprotein complexes in the nucleus. M protein, by interacting with Rae1 and Nup98, would target these complexes to inhibit both transcription and transport of nascent mRNA. Although Nup98 is likely to be important for the M protein-mediated inhibition of nuclear-cytoplasmic RNA transport, it may not be important for the inhibition of host transcription, since silencing Nup98 expression did not affect the inhibition of host transcription by VSV (Figure 5B). Alternatively, the level of silencing of Nup98 may not have been sufficient to have an effect on the inhibition of host transcription by VSV.

The effect of M protein on nuclear accumulation of cellular RNA depends on the cell type and the mRNA target being analyzed. Nuclear accumulation of RNA resulting from the inhibition of transport is most obvious in cells in which M protein has little if any effect on transcription, such as *Xenopus* oocytes [6], [7], [13]. However, in most mammalian cells, there is relatively little net accumulation of constitutively expressed mRNAs relative to pre-existing mRNAs in the nucleus during VSV infection, because their synthesis as well as their transport is inhibited by M protein. This was originally demonstrated in the pulse-chase experiments of Weck and Wagner [20], and is confirmed by our analysis of the distribution of actin mRNA (Figure 7). In contrast to constitutively expressed mRNAs, mRNAs for IL-6 and cJun, which are induced by VSV infection, accumulate in the nucleus, with very little present in the cytoplasm (Figure 7).

Experiments using *in situ* hybridization with oligo-dT have shown an apparent accumulation of total mRNA in the nucleus of VSV-infected cells. However, this result has not been confirmed by an independent approach and may be subject to artifacts such as masking of poly A-containing mRNAs in the cytoplasm of VSV-infected cells as a result of their accumulation in poorly translating ribonucleoprotein particles [47], [48]. Further, not all nuclear-cytoplasmic transport is inhibited by M protein. For example, export of tRNA [6], [7] and RNA bearing constitutive transport element [7] is resistant to the inhibition, as is export of complexes containing hnRNP-A1 and other hnRNPs [49]. Silencing Rae1 expression inhibits export of hnRNP-A1 in VSV-infected cells [49], but has little if any effect on nuclear accumulation of host mRNAs (Figure 7). The level of mRNA in the nucleus reflects a balance of transcription, transport, and turnover. Thus it is possible that silencing Rae1 expression may have an effect on mRNA transport in VSV-infected cells that is balanced by changes in transcription or turnover.

The inhibition of host translation in VSV-infected cells is not due to depletion of host mRNAs from the cytoplasm as a result of the inhibition of host transcription and nuclear-cytoplasmic transport [16], [48], [50]. Instead, the translational apparatus is altered in VSV-infected cells such that translation of pre-existing host mRNAs is inhibited, and only newly appearing mRNAs are translated [16], namely those produced by the viral RNA-dependent RNA polymerase. This alteration of the translation apparatus is correlated with the dephosphorylation of the cap-binding translation factor eIF4E [5], [51]. The results presented here indicate that silencing Rae1 expression has little if any effect on this process (Figure 8), suggesting that other molecular targets are involved.

In summary, the data presented here have addressed the role of host Rae1 in the M protein-mediated inhibition of host gene expression at three different levels. The data support a model in which Rae1 serves as a platform for interaction of M protein with other molecular targets. Our findings also lead us to propose a new function for Rae1 in regulating transcription in VSV- infected cells, as well as providing new insights into the mechanism of VSV-mediated inhibition of host gene expression.

## Methods

### Construction of M protein fusion proteins with glutathione-S-transferase

To construct a wild type (wt) M protein with BamHI and NotI restriction site, the wt M gene in pET21d vector [52] served as a template. A BamHI restriction site was added using the primer 5′GCGGCCGGATCCATGGCTTCCTTAA 3′, and a NotI restriction site was added using reverse primer 5′ GCCCGCGCGGCCGCCTACTCGAGTTTG 3′. The resulting PCR fragment was cleaved and ligated into the vector pGEX-6P-1 (GE Healthcare). The resulting M protein had an N- terminal glutathione-*S-*transferase tag [GST-M]. Mutations within the M gene were made using the Quick change mutagenesis kit (Stratagene). Sequences of all clones were verified through DNA sequencing. The plasmids were transformed into BL21(DE3) pLysS *E.coli* cells.

### Purification of GST-M protein

Day cultures were grown until the cells reached an optical density of A_600 nm_∼0.7 and induced with 200 µM isopropyl-ß-D-thiogalactopyranoside (IPTG) for 3 hours at 37°C. Cells were centrifuged at 4000× g for 20 minutes at 4°C, and pellets were frozen at −20°C until use. Recombinant wt and mutant M proteins with the GST tag were prepared using the protocol described in [13] with a few modifications which are as follows. The cells were lysed in phosphate buffered saline (PBS) with 1% TritonX100 with 1 mM phenyl methylsulfonyl fluoride. Following sonication and centrifugation, the protein was immediately loaded onto glutathione-Sepharose beads (GE Healthcare) and incubated for 1 hour at 4°C. Beads were washed with PBS and used in experiments.

### Infections and lysate preparations of HEK 293 cells

HEK 293 cells were cultured in Dulbecco's modified Eagle's medium with 10% fetal calf serum and 2 mM glutamine. To prepare lysates, the cells were washed with PBS and incubated for 10 minutes on ice in lysis buffer (50 mM Tris pH 8.0, 150 mM NaCl, 15 mM MgCl_2_, and 0.5% NP40) with an EDTA-free protease inhibitor mixture (Roche). Lysates were centrifuged at high speed for 15 minutes at 4°C. The resulting supernatant was collected and frozen at −80°C or used immediately in experiments. For transfections, cells were transfected with 9 µg of plasmid encoding hemagglutinin (HA) epitope tagged Rae1 [a gift from B. Fontoura used in [14]] using the calcium phosphate method. The plasmid encoding HA epitope tagged Rae1 (HA-Rae1) was modified to encode the entire sequence of the HA epitope tag to enhance antibody binding. Twenty four hours post-transfection, the cells were washed with PBS and lysates were prepared as described above. For infecting cells, recombinant wild type VSV with wild type M protein (rwt) or recombinant VSV containing the M51R mutant M protein (rM51R-M) virus stocks were prepared in BHK cells as described [30]. Twenty four hours prior to infection, 1×10^6^ HEK 293 cells were seeded in 100 mm dish. Cells were infected with either rwt or rM51R-M virus at a multiplicity of infection (MOI) of 10 plaque forming units/cell for 6 hours. Following infection, cells were harvested in lysis buffer as described above.

### Binding of cellular proteins to GST-M protein

Wt or mutant GST-M proteins on glutathione beads were incubated in binding buffer (20 mM HEPES pH 7.4, 110 mM potassium acetate, 2 mM MgCl_2_, and 0.1% Tween 20) for 1 hour at 4°C. Lysates from siRNA transfected or untransfected cells (250 µl) were incubated with 25 µl packed volume of GST-M proteins on glutathione beads (500 ng of GST-M protein) suspended in 250 µl of binding buffer at 4°C. Unless otherwise noted, where the incubation time was varied, the proteins were incubated for 1 hour. Bound and unbound fractions were separated by spinning the samples at 4000× g for 2 minutes at 4°C. The bound fraction was washed several times with the binding buffer and analyzed by sodium dodecyl sulfate-polyacrylamide gel electrophoresis (SDS-PAGE) and immunoblotting.

### Immunoprecipitation

Lysates from cells transfected with HA-Rae1 were prepared as described above. The lysates were incubated overnight with 30 µl of HA antibody (Roche). The lysates were incubated with protein A agarose beads (Sigma) prepared in 10 mm Tris pH8.0 for 2 hours at 4°C. The supernatant and pellet fractions were separated by centrifugation at high speed for 5 minutes. The pellet fractions were washed several times with cell lysis buffer and analyzed by sodium dodecyl sulfate-polyacrylamide gel electrophoresis (SDS-PAGE) and immunoblotting.

### Immunoblotting

Proteins were resolved by SDS-PAGE using either 10% Bis-Tris NuPAGE gel (Invitrogen) or 10% Tris-HCl polyacrylamide gels. Following electrophoresis, proteins were transferred onto polyvinylidene difluoride (PVDF) membranes and blocked in Tris buffered saline with 0.02% Tween- 20 (TBS-T) with 5% milk (Difco) or in PBS with 0.05% Tween-20 (PBS-T) and 3% milk. The membranes were probed using primary antibodies to Nup98 (Sigma) or the HA tag (Sigma) or TBP protein (Sigma) prepared in TBS-T with 2.5% milk. Antibody against Rae1 (R2905: Sigma) or M protein (23H12) were prepared in PBS-T with 1% milk. After several washes in either TBS-T or PBS-T, the blots were incubated with respective secondary antibodies linked to horseradish peroxidase (Amersham) used at 1∶10000 in TBS-T with 2.5% milk or PBS-T with 1% milk. The blots were washed in either TBS-T or PBS-T, and proteins were detected using enhanced chemiluminescence substrate (Thermo Scientific). The intensities of the bands were quantified by scanning and analysis using Quantity One software (Bio-Rad).

### Gel filtration chromatography

Lysates from mock-infected cells were concentrated approximately 3-fold to 500 µl using Ultracel −10K (Millipore) before chromatography on size exclusion chromatography. Lysates were chromatographed on a Superdex 200 column (length = 30 cm, diameter = 1 cm) in cell lysis buffer using an FPLC apparatus (Bio-Rad), and thirty 1 ml fractions were collected. The fractions were collected at a flow rate of 0.5 ml per minute at 4°C (approximately 50 min). Equal volumes of fractions were analyzed for the presence of proteins by SDS-PAGE followed by immunoblotting. The standards used to calibrate the column were bovine serum albumin (BSA), ovalbumin, and aldolase prepared in cell lysis buffer without NP40. Gel filtration fractions obtained were incubated with GST-M protein on glutathione beads for 1 hour to obtain bound fractions as described above.

### Sucrose gradient centrifugation

Lysates were overlaid on 5–20% sucrose gradients in cell lysis buffer. Gradients were centrifuged at 35,000 rpm for 18.3 hours at 4°C in a SW41.0 rotor (Beckman Instruments). Twenty fractions of equal volumes were collected from the top of the gradient and analyzed by SDS-PAGE and immunoblotting. The gradient was calibrated with standard proteins of known sedimentation coefficient, BSA (4.4S) and phosphorylase b (8.8S). Sucrose gradient fractions were incubated with GST-M protein on glutathione beads for 14 hours at 4°C to obtain bound and unbound fractions as described above. To immunoprecipitate from sucrose gradient fractions, fractions 2–3, 4–8 and 9–11 were incubated with HA-Rae1 antibody and purified with protein A agarose beads as described above.

### RNA interference and lysate preparation

Rae1 siRNA (D-011482-02, Dharmacon) was used at final concentration of either 5 µM or 10 µM with similar results. Nup98 siRNA (D013078-01, Dharmacon) was used at a final concentration of 5 or 10 µM to achieve similar silencing efficiency. The nontargeting (NT) siRNA whose sequence is scrambled and does not match any sequence on the human genome used was as a control (D-001210-01, Dharmacon). All transient siRNA transfections were carried out in HeLa cells using Hiperfect transfection reagent (Qiagen Corporation) according to the manufacturer's instructions and as described previously [49]. For binding experiments, lysates from silenced cells were prepared at 48 hours post transfection as described above. Silencing of each protein was confirmed by immunoblotting.

### Two-dimensional (2D) gel electrophoresis

Bound and unbound fractions of cell lysates containing endogenous Rae1 or HA-Rae1 after incubation with GST-M protein on glutathione beads were prepared as described above. The bound fraction was washed and the beads were incubated with fresh lysates twice more to increase the amount of bound protein for analysis. The bound fraction was washed after each incubation. The bound fraction was re-suspended in rehydration buffer [8 M urea, 2% chaps, 50 mM dithiothreitol and 0.2% ampholytes (Bio-Rad)]. The unbound fraction was precipitated using 1∶1 ethanol: ether solution and then re-suspended in rehydration buffer. The bound and unbound fractions were incubated for 16 hours at room temperature in an IPG strip pH 3–10, 11 cm (Bio-Rad). The strips were focused in a Protean IEF cell. Following focusing, the strips were run in the second dimension using an 8–16% Tris–HCl gel (Bio-Rad), transferred onto PVDF and probed for Rae1.

### RNA synthesis

HeLa cells were transfected Rae1, Nup98 or NT siRNA, and at 48 hours post transfection, cells were re-seeded at a density of approximately 1×10^6^ cells in 35-mm culture dishes. After 24 hours cells were mock-infected or infected with rwt virus at MOI = 30 in the presence or absence of actinomycin D (5 µg/ml) as described previously [19]. At 6 hours postinfection, cells were labeled with [^3^H]-uridine (100 µCi/ml) for 30 minutes, washed, and harvested in PBS. RNA was precipitated with trichloroacetic acid, and radioactivity was determined by scintillation counting.

### Cytoplasmic and nuclear RNA fractionation

All solutions used for RNA purification were prepared in diethyl pyrocarbonate-treated water. At 72 hours post transfections with Rae1 siRNA or NT siRNA, cells were mock-infected or infected with rwt virus at MOI = 10 for 6 hours. RNA was isolated from the nucleus and cytoplasm as described in [32] with a few modifications which were as follows. After scraping the cells in cold PBS, the pellet was resuspended in lysis buffer (10 mM NaCl, 10 mM Tris-Cl [pH 7.4], 3 mM MgCl_2_) containing 20 mM vanadyl-ribonucleoside complex (Sigma). An equal volume of the same lysis buffer with 10% (vol/vol) deoxycholate and 20% Tween-40 was added to the cells on ice with gentle mixing. Nuclei and cytoplasmic fractions were separated by centrifugation over a sucrose cushion. Supernatant (cytoplasmic) and pellet (nuclear) fractions were analyzed without further manipulation in order to recover equal cell-equivalent amounts of nuclear and cytoplasmic fractions. To normalize the data for RNA recovery, samples were spiked with 3 µg of *E.coli* total mRNA (Ambion) before isolation RNA using TRIzol reagent (Invitrogen).

### Microarrays

At 72 hours post transfection with Rae1 siRNA or NT siRNA, cells were mock-infected or infected with rwt virus at MOI = 30. At 6 h postinfection, total RNA was isolated using TRIzol reagent. Each RNA sample was processed according the manufacturer's protocol (Affymetrix) and hybridized to the Affymetrix Human Genome U219 Array strip representing 20,000 well-characterized human genes. Each chip was scaled to a target intensity of 500, normalized to control probe sets present on each chip, and then expressed as a ratio to the nonspecific background on a per-gene basis. Analysis of data was carried out using Affymetrix Data Mining Tool software (Affymetrix). The intensity values from all of the probe sets on the arrays were log2-transformed and adjusted by systematic variation normalization [53].

### Real time reverse transcription-polymerase chain reaction (RT- PCR)

Oligonucleotide primers and probes were designed and purchased from Sigma-Genosys. Primers for uidA gene (beta-glucuronidase) in *E.coli* were (forward) 5′-AGGTGCACGGGAATATTTCG-3′ and (reverse) 5′- ACGCGTCGGGTCGAGTT-3. The probe for *E.coli* uidA was CCACTGGCGGAAGCAACGCG . Primers for IL-6 gene were (forward) 5′-CCCCCAGGAGAAGATTCCAA- 3′ and (reverse) 5′-TCAATTCGTTCTGAAGAGGTGAGT-3. The probe for IL-6 was ATGTAGCCGCCCCACACAGACAGC. Primers for c Jun gene were (forward) 5′- GCAAAGATGGAAACGACCTTCT- 3′ and (reverse) 5′- GCTCTCGGACGGGAGGAA-3. The probe for c-Jun was TGACGATGCCCTCAACGCCT. The probes for each gene were labeled at the 5′ end with the reporter dye carboxyfluorescein and at the 3′ with the quencher tetramethylrhodamine. The primers and probe sequences for β-actin were as described in [54]. Real time RT-PCR analysis was performed with a TaqMan One-Step RT-PCR Master Mix Reagents kit (Applied Biosystems) as described by the manufacturer using a 25-µl sample volume and 0.25 ng of sample RNA. For actin, IL-6 and c-Jun, 5 µM concentrations of primers, and 2.5 µM concentration of probes were used, and for *E.coli* uidA, 10 µM concentrations of primers and 5 µM concentration of probe were used. TaqMan PCR assays were performed using an ABI 7700 instrument (Applied Biosystems, Foster City, CA) as described [54]. All samples were tested in triplicate. The critical threshold cycle (*C_T_*) is defined as the cycle at which the fluorescence becomes detectable above background and is inversely proportional to the logarithm of the initial number of template molecules. A standard curve was plotted for each primer-probe set with *C_T_* values obtained from amplification of known quantities of plasmid DNA coding for either β-actin or of total *E.coli* mRNA. The standard curves were used to transform *C_T_* values of the experimental samples to the relative number of DNA molecules.

### Determination of the rates of protein synthesis

At 72 hours post transfection with Rae1 siRNA, Nup98 or NT siRNA, cells were mock-infected or infected with rwt virus at MOI = 30 and then labeled with [^35^S] methionine for 10 min at varying times after infection, as described [5]. Lysates were assayed for protein content and equal amounts of protein were resolved on SDS-PAGE gel. Gels were stained with Coomassie blue and were analyzed by phosphorescence imaging (Amersham Biosciences). The intensities of corresponding host and viral protein bands were quantified using ImageQuant software (Molecular Dynamics). For viral protein bands, the background was determined from an equivalently sized region of the gel immediately above the viral protein band. For host protein bands, the regions of the gel devoid of viral proteins between viral L and G, G and N, and P and M proteins were quantified, and similarly sized regions of the image without radioactivity were used as background.

### Luciferase assays

Templates for *in vitro* transcription of M mRNA were generated by linearizing plasmid pSD-M [11], [33] with *SalI*, followed by phenol chloroform extraction and ethanol precipitation. mRNA was transcribed *in vitro* using the mMessage Machine SP6 Kit (Ambion) according to the manufacturer's instructions, and the RNA was precipitated with lithium chloride. HeLa cells were transfected with Rae1 or NT siRNA, and at 48 hours post-transfection were re-seeded at a density of approximately 1×10^6^ in 35-mm culture dishes. After 24 hours, cells were transfected with varying amounts of M mRNA and yeast tRNA to adjust the total RNA to 750 ng, together with pGL3 plasmid DNA (100 ng, Promega) using the Mirus TransIT mRNA reagent. Luciferase activity was determined using Luciferase Assay System (Promega).

### Isolation of chromatin-associated fractions from nuclei

The nuclei from HeLa S2 spinner cells were fractionated essentially as described [36]. All solutions had protease inhibitors added immediately before use. Briefly, cells were pelleted and resuspended in ice-cold RSB buffer (10 mM Tris-Cl [pH 7.4], 0.5 mM MgCl_2_,10 mM KCL). Cell membranes were disrupted in a Dounce homogenizer by 35 strokes of Teflon coated pestle. The integrity of the nuclear membranes were generally intact as monitored by light microscopy. The lysates were overlaid on buffer B (2.3 M sucrose, 50 mM Tris-HCl [pH 7.5], 25 mM KCl, 5 mM MgCl_2_, 2 mM DTT) and spun at 2000 rpm to pellet the nuclei. The pellets were resuspended in 100 µl buffer A (0.25 sucrose, 50 mM Tris-HCl [pH7.5], 25 mM KCl, 5 mM MgCl_2_, 2 mM DTT) and the nuclei were counted and frozen at −80°C until use. 1×10^7^ nuclei were thawed by placing in 30°C water bath and centrifuged at 2500 rpm for 1 minute. The pellet was resuspended by adding 300 µl of lysis buffer (0.1 mM MgCl_2,_ 1 mM DTT, 5 µg/ml of DNaseI and 5 µg/ml of RNaseI) dropwise, and vortexing. Following resuspension, 1.3 ml of extraction buffer (10% sucrose, 20 mM triethanolamine [pH 7.5], 0.1 mM MgCl_2,_ 1 mM DTT) was added dropwise and the pellet was incubated for 15 minutes at room temperature. The resuspended nuclei were underlaid with 500 µl of 30% sucrose cushion (30% sucrose, 20 mM triethanolamine [pH 7.5], 0.1 mM MgCl_2,_ 1 mM DTT) and centrifuged by slowly increasing the speed to 4000 rpm for 10 minutes. The supernatant and pellet fractions were separated and the pellet was resuspended in 300 µl of extraction buffer [pH7.5] dropwise followed by 170 µl of extraction buffer [pH7.5] containing 0.3 mg/ml of heparin. The resuspended nuclei were underlaid on 30% sucrose cushion and centrifuged as before. The process was repeated, with the pellet resuspended in 170 µl of extraction buffer [pH7.5]. The isolated fractions were incubated with GST-M protein or GST for 14 hours suspended in cell lysis buffer and analyzed by SDS-PAGE and immunoblotting. The chromatin-associated fractions and nuclear membrane fractions from uninfected cells were cleanly separated. However, when this procedure was applied to VSV-infected cells, the nuclear membranes appeared to be disrupted during the procedure, perhaps due to greater fragility, so that they were not cleanly separated from the chromatin-associated fractions.

### Accession numbers

Microarray data were deposited in the Gene Expression Omnibus [GEO] database (Accession Number: GSE38866): http://www.ncbi.nlm.nih.gov/geo/query/acc.cgi?acc=GSE38866.

## Supporting Information

Figure S1 (**A**) Recombinant wild type or mutant M proteins were purified on glutathione beads and analyzed by SDS-PAGE and Coomassie Blue staining, demonstrating similar levels of each protein used for experiments. (**B**) HeLa cells were transfected with Rae1 siRNA or with non-targeting (NT) siRNA. 48 hours post-transfection, lysates were analyzed by immunoblots probed for Rae1. Transfection with Rae1 siRNA reduced expression of Rae1, but did not affect expression of a slower migrating protein immunoreactive with Rae1 antibody (arrow). (**C**) Cells were transfected with plasmid DNA encoding HA-Rae1. Cell lysates were incubated with recombinant wild type or mutant M protein GST fusion proteins or GST alone on glutathione beads for 1 hour. Bound and unbound fractions were analyzed by immunoblotting and probed for HA. (**D**) Cells were transfected with plasmid DNA encoding HA-Rae1. At 24 hours post-transfection, cells were infected with rwt virus for 6 hours. Cell lysates were immunoprecipitated using antibody against HA and bound fractions were analyzed by immunoblots probed for M protein.
(TIF)

Figure S2 **Analysis of post-translational modification of Rae1.** Two-dimensional isoelectric focusing/SDS-PAGE was used to analyze bound and unbound fractions of cell lysates containing only endogenous Rae1 (upper panel) or containing HA-Rae1 (lower panel) after incubation with wt GST-M protein on glutathione beads. The fractions were probed for Rae1 and HA. The arrows depict the isoelectric points of known standards subjected to the same conditions.
(TIF)

Figure S3 **Effects of silencing the expression of Nup98 on host and viral protein synthesis.** (**A**) HeLa cells were transfected with Nup98 siRNA or non-targeting (NT) siRNA. 72 hours post-transfection, cells were mock infected or infected with rwt virus for the indicated times and labeled with [^35^S ] methionine for 10 min. Lysates from Nup98 siRNA cells or NT siRNA cells were analyzed by SDS-PAGE and phosphoimaging. Shown is a phosphoimage with the viral proteins indicated on the right. (**B**) Quantification of host protein synthesis expressed as a percentage of mock infected cells. Data shown are the means ± standard deviation of three separate experiments. (**C**) Quantification of viral protein synthesis expressed as a percentage of synthesis at six hours postinfection. Data shown are the means ± standard deviation of three separate experiments.
(TIF)

Table S1 **Effects of silencing the expression of Rae1 on mRNA expression in VSV-infected cells.** HeLa cells were transfected with Rae1 siRNA or NT siRNA, then mock-infected or infected with rwt virus. At 6 h postinfection, total RNA was isolated and analyzed using Affymetrix Human Genome U219 Array strips. Data shown are probe sets that were reproducibly decreased (**A** and **B**) or increased (**C** and **D**) by greater than 3-fold (log_2_ 3 = 1.58) in VSV-infected versus mock-infected siNT cells (**A** and **C**) or siRae1 cells (**B** and **D**). The selection criteria were that the pooled variance in the probe set in repeat experiments gave p<0.005 in comparing VSV-infected to mock-infected cells. In siNT cells, 880 probe sets met this criterion, of which 17 were decreased >3-fold (**A**) and 12 were increased >3-fold (**C**). In siRae1 cells, 970 probe sets met this criterion, of which 5 were decreased >3-fold (**B**) and 41 were increased >3-fold (**D**). The selection of a 3-fold difference as a criterion for this table was based on a comparison of mock-infected siRae1 versus siNT cells. In this comparison, 713 probe sets had p<0.005. Of these only 3 were decreased >3-fold (**E**), including Rae1 itself.
(DOC)
