# Supplementary figures and images for: Complexes of Vesicular Stomatitis Virus Matrix Protein with Host Rae1 and Nup98 Involved in Inhibition of Host Transcription
Source: PLoS Pathog. 2012 Sep 27;8(9):e1002929. doi: 10.1371/journal.ppat.1002929 (PMC3460625; doi:10.1371/journal.ppat.1002929)

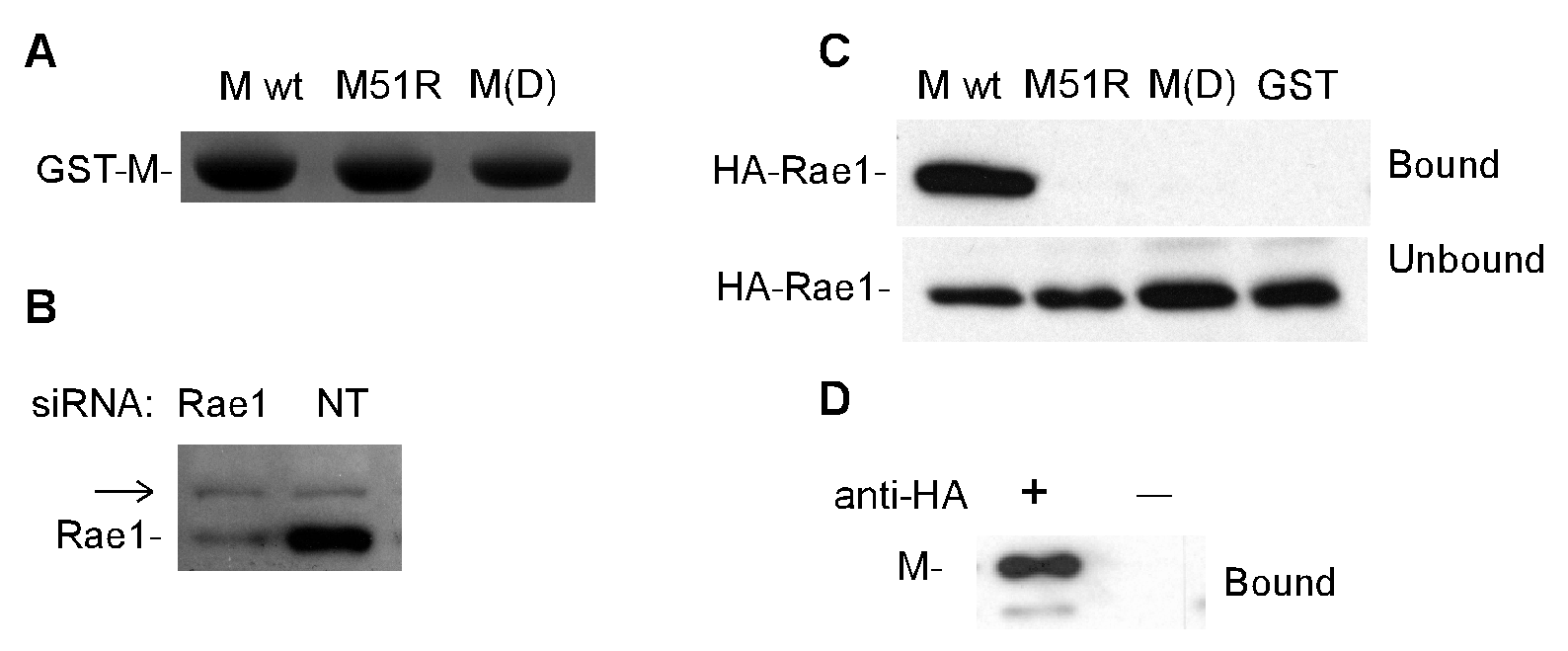

Supplement: Figure S1 — (A) Recombinant wild type or mutant M proteins were purified on glutathione beads and analyzed by SDS-PAGE and Coomassie Blue staining, demonstrating similar levels of each protein used for experiments. (B) HeLa cells were transfected with Rae1 siRNA or with non-targeting (NT) siRNA. 48 hours post-transfection, lysates were analyzed by immunoblots probed for Rae1. Transfection with Rae1 siRNA reduced expression of Rae1, but did not affect expression of a slower migrating protein immunoreactive with Rae1 antibody (arrow). (C) Cells were transfected with plasmid DNA encoding HA-Rae1. Cell lysates were incubated with recombinant wild type or mutant M protein GST fusion proteins or GST alone on glutathione beads for 1 hour. Bound and unbound fractions were analyzed by immunoblotting and probed for HA. (D) Cells were transfected with plasmid DNA encoding HA-Rae1. At 24 hours post-transfection, cells were infected with rwt virus for 6 hours. Cell lysates were immunoprecipitated using antibody against HA and bound fractions were analyzed by immunoblots probed for M protein. (TIF) [file ppat.1002929.s001.tif]

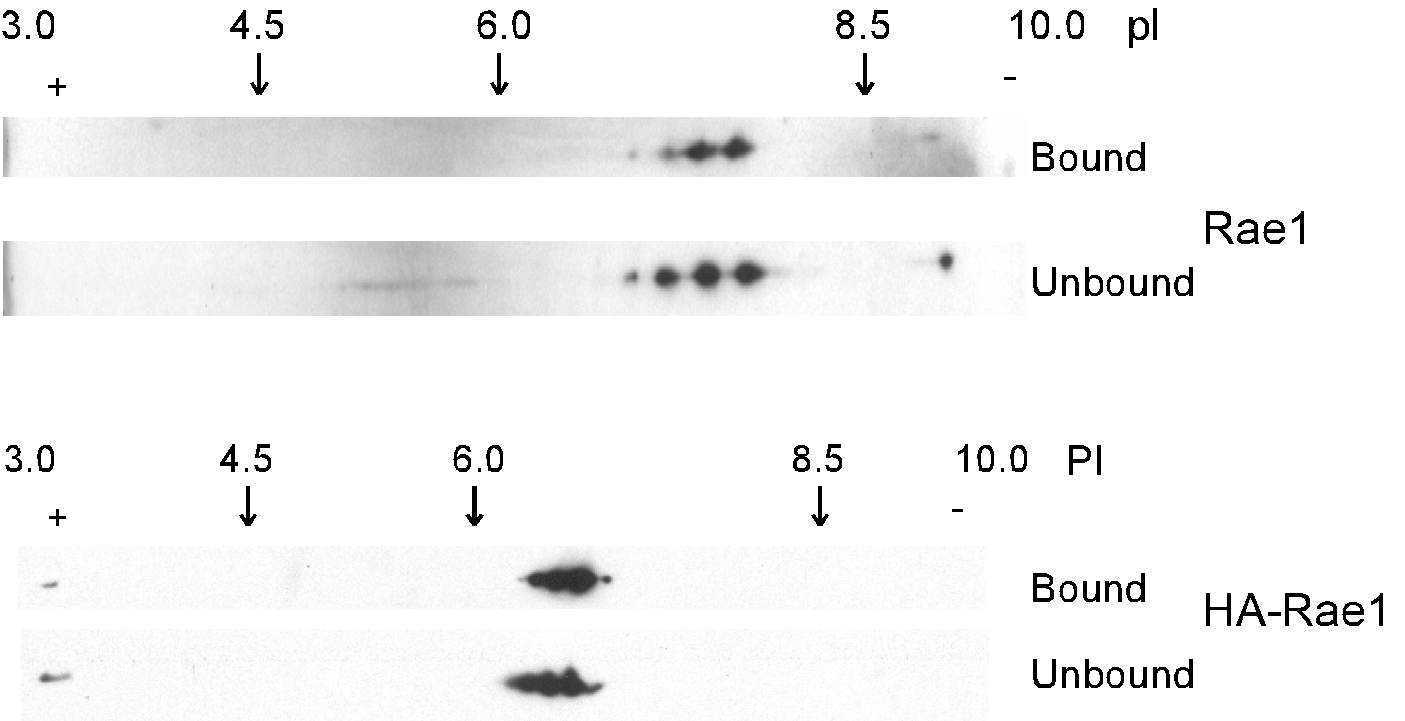

Supplement: Figure S2 — Analysis of post-translational modification of Rae1. Two-dimensional isoelectric focusing/SDS-PAGE was used to analyze bound and unbound fractions of cell lysates containing only endogenous Rae1 (upper panel) or containing HA-Rae1 (lower panel) after incubation with wt GST-M protein on glutathione beads. The fractions were probed for Rae1 and HA. The arrows depict the isoelectric points of known standards subjected to the same conditions. (TIF) [file ppat.1002929.s002.tif]

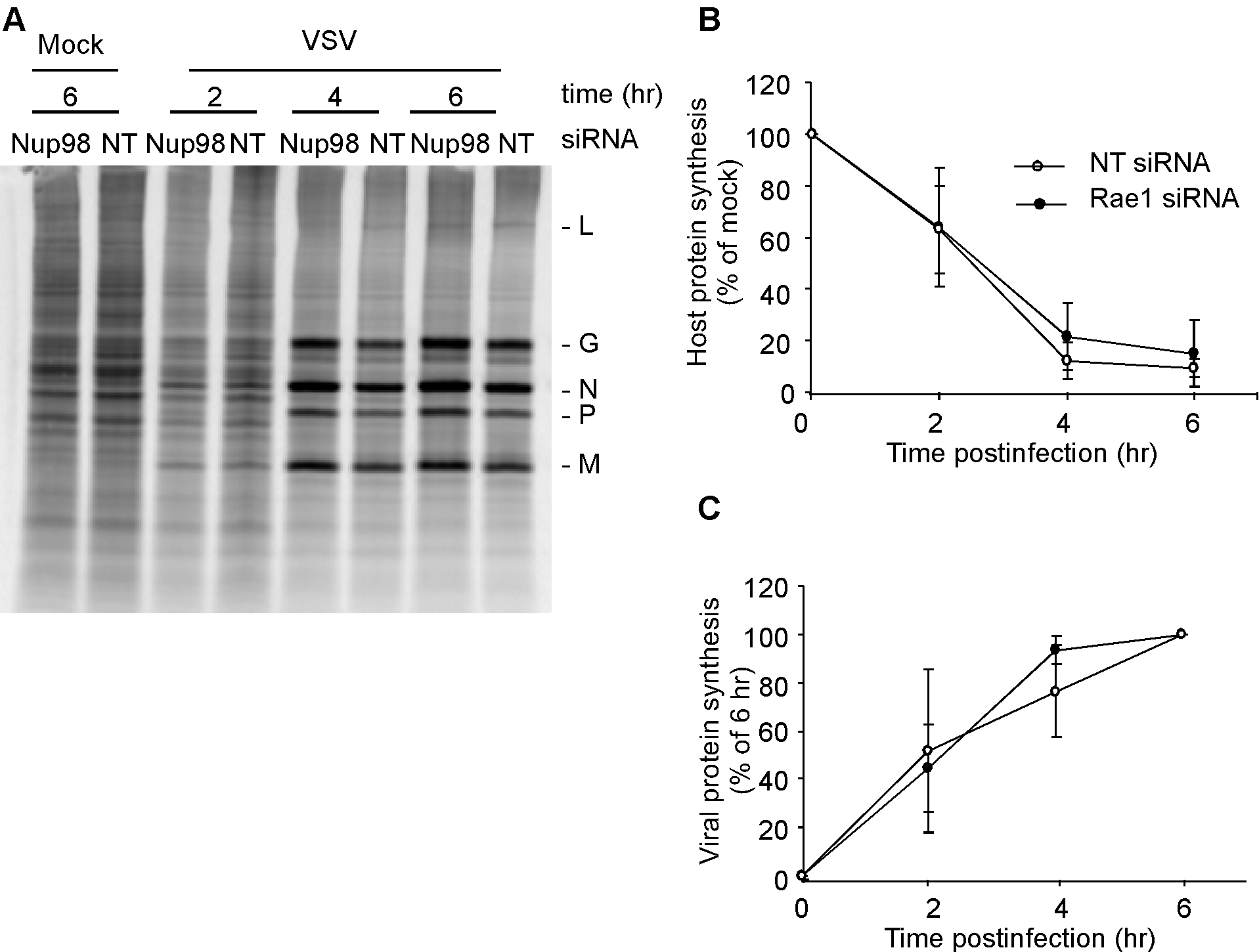

Supplement: Figure S3 — Effects of silencing the expression of Nup98 on host and viral protein synthesis. (A) HeLa cells were transfected with Nup98 siRNA or non-targeting (NT) siRNA. 72 hours post-transfection, cells were mock infected or infected with rwt virus for the indicated times and labeled with [35S ] methionine for 10 min. Lysates from Nup98 siRNA cells or NT siRNA cells were analyzed by SDS-PAGE and phosphoimaging. Shown is a phosphoimage with the viral proteins indicated on the right. (B) Quantification of host protein synthesis expressed as a percentage of mock infected cells. Data shown are the means ± standard deviation of three separate experiments. (C) Quantification of viral protein synthesis expressed as a percentage of synthesis at six hours postinfection. Data shown are the means ± standard deviation of three separate experiments. (TIF) [file ppat.1002929.s003.tif]
