# Supplementary material for: Complexes of Vesicular Stomatitis Virus Matrix Protein with Host Rae1 and Nup98 Involved in Inhibition of Host Transcription
Source: PLoS Pathog. 2012 Sep 27;8(9):e1002929. doi: 10.1371/journal.ppat.1002929 (PMC3460625; doi:10.1371/journal.ppat.1002929)
Supplement: Table S1 — Effects of silencing the expression of Rae1 on mRNA expression in VSV-infected cells. HeLa cells were transfected with Rae1 siRNA or NT siRNA, then mock-infected or infected with rwt virus. At 6 h postinfection, total RNA was isolated and analyzed using Affymetrix Human Genome U219 Array strips. Data shown are probe sets that were reproducibly decreased (A and B) or increased (C and D) by greater than 3-fold (log2 3 = 1.58) in VSV-infected versus mock-infected siNT cells (A and C) or siRae1 cells (B and D). The selection criteria were that the pooled variance in the probe set in repeat experiments gave p<0.005 in comparing VSV-infected to mock-infected cells. In siNT cells, 880 probe sets met this criterion, of which 17 were decreased >3-fold (A) and 12 were increased >3-fold (C). In siRae1 cells, 970 probe sets met this criterion, of which 5 were decreased >3-fold (B) and 41 were increased >3-fold (D). The selection of a 3-fold difference as a criterion for this table was based on a comparison of mock-infected siRae1 versus siNT cells. In this comparison, 713 probe sets had p<0.005. Of these only 3 were decreased >3-fold (E), including Rae1 itself. (DOC) [file ppat.1002929.s004.doc]

**Table S1. Effects of silencing the expression of Rae1 on mRNA expression in VSV-infected cells.**

| **A. Decreased >3-fold in siNT cells** | | | | | | | |
| --- | --- | --- | --- | --- | --- | --- | --- |
| ProbeSet | Gene Symbol | siNT  VSV/mock  (log_2_) | p value |  | siRae1  VSV/mock  (log_2_) | p value | Gene Title |
| 11737912_s_at | TM2D3 | -1.60 | 0.00013 |  | -0.66 | 0.06532 | TM2 domain containing 3 |
| 11725518_at | FRAT2 | -1.63 | 0.00384 |  | -1.02 | 0.01245 | frequently rearranged in advanced T-cell lymphomas 2 |
| 11717633_at | KLHL9 | -1.64 | 0.00219 |  | -0.75 | 0.04581 | kelch-like 9 (Drosophila) |
| 11759602_a_at | C1orf63 | -1.68 | 0.00169 |  | -0.61 | 0.05224 | chromosome 1 open reading frame 63 |
| 11729801_at | EIF1AD | -1.68 | 0.00281 |  | -0.15 | 0.19108 | eukaryotic translation initiation factor 1A domain containing |
| 11716629_at | IER5 | -1.70 | 0.00075 |  | -0.37 | 0.02244 | immediate early response 5 |
| 11730394_at | ZNF552 | -1.71 | 0.00272 |  | -1.60 | 0.02575 | zinc finger protein 552 |
| 11719285_a_at | PEX11B | -1.73 | 0.00408 |  | -1.41 | 0.00597 | peroxisomal biogenesis factor 11 beta |
| 11745535_a_at | CRB1 | -1.74 | 0.00007 |  | -1.76 | 0.00226 | crumbs homolog 1 (Drosophila) |
| 11723209_s_at | KBTBD6 | -1.85 | 0.00243 |  | -1.21 | 0.00509 | kelch repeat and BTB (POZ) domain containing 6 |
| 11727958_a_at | ING1 | -1.86 | 0.00446 |  | -0.69 | 0.00123 | inhibitor of growth family, member 1 |
| 11756100_s_at | TMEM60 | -1.92 | 0.00278 |  | -1.74 | 0.00679 | transmembrane protein 60 |
| 11719562_at | SOX4 | -1.96 | 0.00489 |  | -0.98 | 0.00184 | SRY (sex determining region Y)-box 4 |
| 11754525_s_at | RNF146 | -2.07 | 0.00087 |  | -0.60 | 0.01361 | ring finger protein 146 |
| 11758582_s_at | SIX4 | -2.16 | 0.00230 |  | -1.05 | 0.01596 | SIX homeobox 4 |
| 11744928_x_at | SIAH1 | -2.18 | 0.00079 |  | -1.08 | 0.01530 | seven in absentia homolog 1 (Drosophila) |
| 11729711_at | MARS2 | -2.48 | 0.00184 |  | -1.52 | 0.00434 | methionyl-tRNA synthetase 2, mitochondrial |
|  | | | | | | | |
| **B. Decreased >3-fold in siRae1 cells** | | | | | | | |
| ProbeSet | Gene Symbol | siRae1  VSV/mock  (log_2_) | p value |  | siNT  VSV/mock  (log_2_) | p value | Gene Title |
| 11718540_at | C7orf23 | -1.60 | 0.00436 |  | -1.28 | 0.00749 | chromosome 7 open reading frame 23 |
| 11757415_s_at | SLC5A3 | -1.65 | 0.00335 |  | -1.56 | 0.00924 | solute carrier family 5 (sodium/myo-inositol cotransporter), member 3 |
| 11745535_a_at | CRB1 | -1.76 | 0.00226 |  | -1.74 | 0.00007 | crumbs homolog 1 (Drosophila) |
| 11763395_a_at | ZC3HAV1L | -1.81 | 0.00008 |  | -1.17 | 0.05229 | zinc finger CCCH-type, antiviral 1-like |
| 11729121_a_at | MKKS | -1.87 | 0.00279 |  | -0.92 | 0.07141 | McKusick-Kaufman syndrome |
|  | | | | | | | |
| **C. Increased >3-fold in siNT cells** | | | | | | | |
| ProbeSet | Gene Symbol | siNT  VSV/mock  (log_2_) | p value |  | siRae1  VSV/mock  (log_2_) | p value | Gene Title |
| 11734797_x_at | HIST1H2BG | 2.60 | 0.00328 |  | 0.89 | 0.02166 | histone cluster 1, H2bg |
| 11734796_s_at | HIST1H2BG | 2.60 | 0.00047 |  | 0.96 | 0.00490 | histone cluster 1, H2bg |
| 11715514_a_at | HERPUD1 | 2.20 | 0.01567 |  | 3.27 | 0.00906 | homocysteine-inducible, endoplasmic reticulum stress-inducible, ubiquitin-like domain member 1 |
| 11741510_a_at | HERPUD1 | 2.18 | 0.02194 |  | 3.37 | 0.01078 | homocysteine-inducible, endoplasmic reticulum stress-inducible, ubiquitin-like domain member 1 |
| 11735775_at | HIST1H4K | 2.18 | 0.01592 |  | 1.99 | 0.06301 | histone cluster 1, H4k |
| 11718397_s_at | JUN | 2.08 | 0.00475 |  | 0.54 | 0.04325 | jun oncogene |
| 11749257_a_at | HERPUD1 | 2.06 | 0.00805 |  | 3.25 | 0.01128 | homocysteine-inducible, endoplasmic reticulum stress-inducible, ubiquitin-like domain member 1 |
| 11753421_a_at | CITED2 | 1.93 | 0.01106 |  | 0.92 | 0.00180 | Cbp/p300-interacting transactivator, with Glu/Asp-rich carboxy-terminal domain, 2 |
| 11718394_at | JUN | 1.81 | 0.00470 |  | 1.16 | 0.00477 | jun oncogene |
| 11726024_at | EDN1 | 1.69 | 0.01210 |  | 1.80 | 0.00885 | endothelin 1 |
| 11749169_s_at | KRT6A /// KRT6B /// KRT6C | 1.62 | 0.00220 |  | 1.18 | 0.02790 | keratin 6A /// keratin 6B /// keratin 6C |
| 11736556_s_at | SLC2A14 /// SLC2A3 | 1.62 | 0.01076 |  | 1.22 | 0.01559 | solute carrier family 2 (facilitated glucose transporter), member 14 /// solute carrier family 2 (facilitated glucose transporter), member 3 |
|  | | | | | | | |
| **D. Increased >3-fold in siRae1 cells** | | | | | | | |
| ProbeSet | Gene Symbol | siRae1  VSV/mock  (log_2_) | p value |  | siNT  VSV/mock  (log_2_) | p value | Gene Title |
| 11721874_at | IFIT2 | 4.06 | 0.00376 |  | 1.51 | 0.10532 | interferon-induced protein with tetratricopeptide repeats 2 |
| 11746463_a_at | IL6 | 3.71 | 0.00139 |  | 2.39 | 0.00902 | interleukin 6 (interferon, beta 2) |
| 11744128_x_at | CXCL2 | 3.66 | 0.00180 |  | 1.32 | 0.00504 | chemokine (C-X-C motif) ligand 2 |
| 11744127_at | CXCL2 | 3.57 | 0.00149 |  | 1.43 | 0.01332 | chemokine (C-X-C motif) ligand 2 |
| 11741510_a_at | HERPUD1 | 3.37 | 0.00095 |  | 2.18 | 0.00458 | homocysteine-inducible, endoplasmic reticulum stress-inducible, ubiquitin-like domain member 1 |
| 11721873_at | IFIT2 | 3.31 | 0.00281 |  | 0.83 | 0.23069 | interferon-induced protein with tetratricopeptide repeats 2 |
| 11715514_a_at | HERPUD1 | 3.27 | 0.00085 |  | 2.20 | 0.00323 | homocysteine-inducible, endoplasmic reticulum stress-inducible, ubiquitin-like domain member 1 |
| 11749257_a_at | HERPUD1 | 3.25 | 0.00107 |  | 2.06 | 0.00190 | homocysteine-inducible, endoplasmic reticulum stress-inducible, ubiquitin-like domain member 1 |
| 11756820_a_at | IFIT1 | 2.78 | 0.00436 |  | 0.42 | 0.40058 | interferon-induced protein with tetratricopeptide repeats 1 |
| 11752993_a_at | DUSP1 | 2.77 | 0.00360 |  | 1.27 | 0.03930 | dual specificity phosphatase 1 |
| 11715766_a_at | DUSP1 | 2.66 | 0.00115 |  | 1.04 | 0.02219 | dual specificity phosphatase 1 |
| 11719344_a_at | ATF3 | 2.61 | 0.00070 |  | 1.05 | 0.02741 | activating transcription factor 3 |
| 11734659_a_at | FOS | 2.58 | 0.00031 |  | 1.21 | 0.02304 | FBJ murine osteosarcoma viral oncogene homolog |
| 11732276_x_at | CCL5 | 2.43 | 0.00282 |  | 0.99 | 0.04542 | chemokine (C-C motif) ligand 5 |
| 11715795_at | MANF | 2.28 | 0.00042 |  | 1.30 | 0.00813 | mesencephalic astrocyte-derived neurotrophic factor |
| 11717367_at | ZC3HAV1 | 2.24 | 0.00119 |  | 0.95 | 0.09730 | zinc finger CCCH-type, antiviral 1 |
| 11717863_a_at | DUSP5 | 2.17 | 0.00458 |  | -0.22 | 0.15780 | dual specificity phosphatase 5 |
| 11758198_s_at | DDIT3 | 2.14 | 0.00253 |  | 1.89 | 0.01220 | DNA-damage-inducible transcript 3 |
| 11744000_a_at | NFKBIA | 2.02 | 0.00349 |  | 0.19 | 0.44497 | nuclear factor of kappa light polypeptide gene enhancer in B-cells inhibitor, alpha |
| 11716093_a_at | KLF6 | 2.02 | 0.00215 |  | 0.55 | 0.18974 | Kruppel-like factor 6 |
| 11720298_at | CXCL10 | 1.97 | 0.00136 |  | 0.45 | 0.14296 | chemokine (C-X-C motif) ligand 10 |
| 11717366_at | ZC3HAV1 | 1.97 | 0.00498 |  | 0.70 | 0.09876 | zinc finger CCCH-type, antiviral 1 |
| 11725983_at | BHLHE40 | 1.97 | 0.00072 |  | -0.20 | 0.00205 | basic helix-loop-helix family, member e40 |
| 11742752_a_at | ETS2 | 1.96 | 0.00127 |  | 0.48 | 0.02250 | v-ets erythroblastosis virus E26 oncogene homolog 2 (avian) |
| 11753661_a_at | ID1 | 1.93 | 0.00384 |  | -0.74 | 0.04780 | inhibitor of DNA binding 1, dominant negative helix-loop-helix protein |
| 11743036_s_at | SAT1 | 1.93 | 0.00171 |  | 0.63 | 0.00424 | spermidine/spermine N1-acetyltransferase 1 |
| 11757894_x_at | NFKBIA | 1.91 | 0.00313 |  | 0.01 | 0.94762 | nuclear factor of kappa light polypeptide gene enhancer in B-cells inhibitor, alpha |
| 11716095_s_at | KLF6 | 1.91 | 0.00179 |  | 0.52 | 0.19114 | Kruppel-like factor 6 |
| 11725930_s_at | HSPA5 | 1.90 | 0.00106 |  | 1.26 | 0.01894 | heat shock 70kDa protein 5 (glucose-regulated protein, 78kDa) |
| 11726023_a_at | EDN1 | 1.89 | 0.00118 |  | 1.98 | 0.03132 | endothelin 1 |
| 11718940_a_at | TNFAIP3 | 1.88 | 0.00185 |  | 0.87 | 0.01637 | tumor necrosis factor, alpha-induced protein 3 |
| 11731407_x_at | IFIT3 | 1.84 | 0.00484 |  | 0.04 | 0.95105 | interferon-induced protein with tetratricopeptide repeats 3 |
| 11753632_x_at | ATF3 | 1.83 | 0.00185 |  | 0.50 | 0.22766 | activating transcription factor 3 |
| 11726024_at | EDN1 | 1.80 | 0.00271 |  | 1.69 | 0.00421 | endothelin 1 |
| 11759322_at | BCL6 | 1.78 | 0.00031 |  | 0.67 | 0.13149 | B-cell CLL/lymphoma 6 |
| 11717368_x_at | ZC3HAV1 | 1.77 | 0.00470 |  | 0.32 | 0.44447 | zinc finger CCCH-type, antiviral 1 |
| 11745878_x_at | NFKBIA | 1.75 | 0.00472 |  | -0.02 | 0.90131 | nuclear factor of kappa light polypeptide gene enhancer in B-cells inhibitor, alpha |
| 11715757_a_at | RGS2 | 1.72 | 0.00024 |  | -0.21 | 0.76486 | regulator of G-protein signaling 2, 24kDa |
| 11735776_x_at | HIST1H4K | 1.63 | 0.00373 |  | 0.48 | 0.02578 | histone cluster 1, H4k |
| 11716710_a_at | ADM | 1.61 | 0.00298 |  | 1.26 | 0.00230 | Adrenomedullin |
| 11746779_a_at | NCOA7 | 1.59 | 0.00436 |  | 1.47 | 0.02610 | nuclear receptor coactivator 7 |
|  |  |  |  |  |  |  |  |
| **E. Decreased >3-fold in mock-infected siRae1 versus siNT cells** | | | | | | | |
| ProbeSet | Gene Symbol | siRae1  mock/siNT mock  (log_2_) | p value |  |  |  | Gene Title |
| 11718766_at | PRSS23 | -1.63212 | 0.00345 |  |  |  | protease, serine, 23 |
| 11756072_s_at | SAA1 /// SAA2 | -2.13505 | 0.00118 |  |  |  | serum amyloid A1 /// serum amyloid A2 |
| 11716236_s_at | RAE1 | -2.6168 | 0.00213 |  |  |  | RAE1 RNA export 1 homolog (S. pombe) |
